# Phylogenetic analysis and comparative genomics of SARS-CoV-2 from survivor and non-survivor COVID-19 patients in Cordoba, Argentina

**DOI:** 10.1186/s12864-022-08756-6

**Published:** 2022-07-14

**Authors:** Nadia B. Olivero, Ana S. Gonzalez-Reiche, Viviana E. Re, Gonzalo M. Castro, María B. Pisano, Paola Sicilia, María G. Barbas, Zenab Khan, Adriana van de Guchte, Jayeeta Dutta, Paulo R. Cortes, Mirelys Hernandez-Morfa, Victoria E. Zappia, Lucia Ortiz, Ginger Geiger, Daniela Rajao, Daniel R. Perez, Harm van Bakel, Jose Echenique

**Affiliations:** 1 Departamento de Bioquimica Clinica, CIBICI (CONICET), Facultad de Ciencias Quimicas, Universidad Nacional de Cordoba. Medina Allende esq. Haya de la Torre, Ciudad Universitaria, X5000HUA Córdoba, Provincia de Córdoba Argentina; 2 Department of Genetics and Genomics Sciences, Icahn School of Medicine at Mount Sinai, New York, NY USA; 3 Instituto de Virologia “Dr. J. M. Vanella”- InViV (CONICET), Facultad de Ciencias Medicas, Universidad Nacional de Córdoba, Córdoba, Argentina; 4 Departamento Laboratorio Central, Ministerio de Salud de la Provincia de Córdoba, Córdoba, Argentina; 5 Secretaria de Prevención y Promoción de la Salud, Ministerio de Salud de la Provincia de Córdoba, Córdoba, Argentina; 6 Department of Population Health, College of Veterinary Medicine, University of Georgia, Athens, GA USA; 7 Department of Pathology, Molecular, and Cell-Based Medicine, Icahn School of Medicine at Mount Sinai, New York, NY 10029 USA; 8 Icahn Genomics Institute, Icahn School of Medicine at Mount Sinai, New York, NY 10029 USA

**Keywords:** COVID-19, Severe acute respiratory syndrome coronavirus 2, SARS-CoV-2, Infectious diseases, Sequencing, Molecular epidemiology, Genomes, Comparative genomics

## Abstract

**Background:**

The SARS-CoV-2 virus is responsible for the COVID-19 pandemic. To better understand the evolution of SARS-CoV-2 early in the pandemic in the Province of Cordoba, Argentina, we performed a comparative genomic analysis of SARS-CoV-2 strains detected in survivors and non-survivors of COVID-19. We also carried out an epidemiological study to find a possible association between the symptoms and comorbidities of these patients with their clinical outcomes.

**Results:**

A representative sampling was performed in different cities in the Province of Cordoba. Ten and nine complete SARS-CoV-2 genomes were obtained by next-generation sequencing of nasopharyngeal specimens from non-survivors and survivors, respectively. Phylogenetic and phylodynamic analyses revealed multiple introductions of the most common lineages in South America, including B.1, B.1.1.1, B.1.499, and N.3. Fifty-six mutations were identified, with 14% of those in common between the non-survivor and survivor groups. Specific SARS-CoV-2 mutations for survivors constituted 25% whereas for non-survivors they were 41% of the repertoire, indicating partial selectivity. The non-survivors’ variants showed higher diversity in 9 genes, with a majority in Nsp3, while the survivors’ variants were detected in 5 genes, with a higher incidence in the Spike protein. At least one comorbidity was present in 60% of non-survivor patients and 33% of survivors. Age 75–85 years (*p* = 0.018) and hospitalization (*p* = 0.019) were associated with non-survivor patients. Related to the most common symptoms, the prevalence of fever was similar in both groups, while dyspnea was more frequent among non-survivors and cough among survivors.

**Conclusions:**

This study describes the association of clinical characteristics with the clinical outcomes of survivors and non-survivors of COVID-19 patients, and the specific mutations found in the genome sequences of SARS-CoV-2 in each patient group. Future research on the functional characterization of novel mutations should be performed to understand the role of these variations in SARS-CoV-2 pathogenesis and COVID-19 disease outcomes. These results add new genomic data to better understand the evolution of the SARS-CoV-2 variants that spread in Argentina during the first wave of the COVID-19 pandemic.

**Supplementary Information:**

The online version contains supplementary material available at 10.1186/s12864-022-08756-6.

## Background

In December 2019, deep sequencing analysis of lower respiratory tract samples from patients with coronavirus disease 2019 (COVID-19) led to the discovery of the novel human coronavirus associated with severe acute respiratory syndrome, known as Severe Acute Respiratory Syndrome coronavirus 2 (SARS-CoV-2), in Wuhan, Hubei Province, China [1, 2].

SARS-CoV-2 is an enveloped virus with a nonsegmented, single-stranded RNA genome that belongs to the Coronaviridae family. SARS-CoV-2 has 10 open reading frames (ORFs) that code for non-structural, structural, and accessory proteins [3].

In general, RNA viruses have high mutation rates that correlate with their adaptation and evolution, traits considered essential for their spread [4]. Despite SARS-CoV-2 being at the low end of that spectrum due to its RNA proofreading capacity, it has clearly shown adaptability and the capacity to generate variants during its worldwide spread. The COVID-19 pandemic was officially declared by the World Health Organization (WHO) on March 12th, 2020 [5]. Two months after the first case was reported in China, the first case in Buenos Aires, Argentina, was confirmed on March 3rd, 2020 [6]. Since then, the number of confirmed SARS-CoV-2 cases has reached 9.3 million (April 30th, 2022) [7].

Despite very strict lockdowns imposed by the national government, Argentina had the first peak of COVID-19 cases between September and November 2020, with > 18,000 positive cases a day. The Province of Cordoba is located in the North Central region of the country and is one of the most populated areas. Its capital, Cordoba, is among the three largest cities in Argentina, along with Buenos Aires and Rosario, in the provinces of Buenos Aires and Santa Fe, respectively. In Argentina, the province of Cordoba has one of the highest rates of COVID-19, with extensive pockets of persistent outbreaks.

This work reports SARS-CoV-2 genome sequences of the first 19 COVID-19 survivors and non-survivors in Cordoba during the first wave of the pandemic in September 2020. Phylogenetic comparison with whole-genome sequences reported from other countries revealed different lineages and potential arrival routes of SARS-CoV-2. A comparative genomic study permitted the identification of specific mutations for survivors and non-survivors, which do not necessarily correlate with the severity of clinical illness. In addition, we found an association between the symptoms and comorbidities of these COVID-19 patients with their clinical outcomes. This work allowed us to highlight the SARS-CoV-2 variants circulating among the population of the Central Region of Argentina.

## Results

### Demographic and clinical characteristics

In this retrospective, multicenter study, 19 complete SARS-CoV-2 genomes were obtained by sequencing clinical specimens from survivors (*n* = 9) and non-survivors (*n* = 10) COVID-19 patients with comprehensive medical records from different cities in the Province of Cordoba, Argentina (Table 1; Fig. S1). COVID-19 diagnoses followed the World Health Organization’s interim guidance [8]. We found no differences in the Ct values for SARS-CoV-2 qRT-PCR diagnosis between survivors and non-survivors (Table 1).

**Table 1:** Epidemiological data of the genome sequences of SARS-CoV-2 2obtained from COVID-19 patients in Cordoba

Genebank ID	Isolation site	N Ct value	Age	Gender	Symp- toms	Comor- bidities	Hospita- lization	Disease status	Pangolin lineage (Nextstrain Clade)	Putative Origin (% identity)
MW633892.1	Cordoba City	23.1	17	F	Fv, C, O	Nc	N	S	N.3 (20B)	Brazil (99.88)
MW633894.1	Hernando	26.5	67	M	Fv, C, O	Db, Ht, Rd.	N	S	B.1.499 (B.1)	Brazil (99.96)
MW633895.1	Villa Ascasubi	22.2	61	F	C	Nc	N	S	B.1.499 (B.1)	USA (99.98)
MW633897.1	Villa Maria	23.0	86	F	Fv, D	Nd	Y	S	N.3 (20B)	Chile (99.97)
MW633898.1	Villa Maria	21.3	93	F	Fv, C	Ht	N	S	B.1 (B.1)	USA (99.98)
MW633902.1	Villa Maria	25.0	56	F	Fv, C, H	Ht	N	S	B.1.499 (B.1)	UK (99.98)
MW633904.1	Dean Funes	28.3	57	M	D	Rd	Y	S	B.1.1.33 (20B)	USA (99.98)
MW633905.1	Cordoba City	25.0	68	M	Fv, C	Db, Ht, Cd	N	S	N.3 (20B)	Chile (99.99)
MW633908.1	Hernando	26.5	68	F	Fv, C, O	Ht, Cd	Y	S	B.1.499 (B.1)	UK (99.97)
**MW633891.1**	**Cordoba City**	**24.0**	**77**	**M**	**Fv, D**	**Nc**	**Y**	**NS**	**B.1.499 (B.1)**	**USA (99.97)**
**MW633893.1**	**Cordoba City**	**22.0**	**76**	**M**	**Fv, D**	**Db, Ht**	**Y**	**NS**	**N.3 (20B)**	**Brazil (99.96**
**MW633896.1**	**Cordoba City**	**21.6**	**73**	**M**	**Fv**	**Db, Ht, Cd**	**Y**	**NS**	**N.3 (20B)**	**Chile (99.98)**
**MW633899.1**	**Hca. Renanco**	**24.6**	**78**	**F**	**Fv**	**Db, Ht**	**N**	**NS**	**B.1.499 (B.1)**	**USA (99.99)**
**MW633900.1**	**Hernando**	**26.0**	**63**	**M**	**Fv, C, D**	**Ht, Rd**	**Y**	**NS**	**B.1.499 (B.1)**	**USA (99.97)**
**MW633901.1**	**Cordoba**	**23.1**	**66**	**F**	**D**	**Rd**	**Y**	**NS**	**B.1.1.1 (20B)**	**UK (99.96)**
**MW633903.1**	**Pblo. Italiano**	**23.2**	**85**	**M**	**Fv, C, D**	**Rd**	**Y**	**NS**	**B.1.499 (B.1)**	**USA (99.99)**
**MW633906.1**	**Cordoba City**	**19.7**	**85**	**F**	**C, D**	**Db, Ht**	**Y**	**NS**	**N.3 (20B)**	**Brazil (99.89)**
**MW633907.1**	**Rio Tercero**	**23.2**	**78**	**F**	**A**	**Db, Rd**	**Y**	**NS**	**B.1.499 (B.1)**	**USA (99.98)**
**MW633909.1**	**Cordoba City**	**24.1**	**59**	**F**	**D**	**Rd**	**Y**	**NS**	**N.3 (20B)**	**USA (99.97)**

References: *M* male, *F* female, *S* survivors, *NS* non-survivors, *A* asymptomatic, *Fv* Fever, *C* cough, *D* dyspnea, *O* odynophagia, *H* headache, *Db* diabetes, *Ht* hypertension, *Rd*. respiratory disease, *Nd* neurological disease, *Cd* cardiac disease, *Nc* no comorbidities. Data of non-survivor patients are in bold

Non-survivor COVID-19 patients had a median age of 74.0 years (range 59–85 years), whereas COVID-19 survivors had a median age of 63.6 years (range 17–93 years). The group of non-survivors aged 76 to 85 years was significantly enriched compared to survivors (*p* = 0.018; Table 2). Most survivors (66%) were female (Table 2), while non-survivors had greater hospitalization rates (*p =* 0.019) (Table 2).

**Table 2:** Clinical summary of the COVID-19 patients

Groups		Total (n = 19)	Survivors (n = 9)	Non survivors (n = 10)	***p***-value^**1**^
Gender F|M		11|8	6|3	5|5	0.650
Age	≤55	1(5.26%)	1 (11.11%)	0 (0%)	0.474
	56–65	5(26.31%)	3 (33.33%)	2 (20%)	0.628
	66–75	5(26.31%)	3 (33.33%)	2 (20%)	0.628
	76–85	6 (31.58%)	0 (0%)	6 (60%)	**0.018**
	> 85	2(10.53%)	0 (0%)	2 (20%)	0.210
Ct qPCR		23.2	23.2	24.05	0.269^2^
**Symptoms**					
Fever		13 (68.42%)	7 (77.78%)	6 (60%)	0.628
Cough		10 (52.63%)	7 (77.78%)	3 (30%)	0.069
Dyspnea		9 (47.36%)	2 (22.22%)	7 (70%)	0.069
Odynophagia		3 (15.79%)	3 (33.33%)	0 (0%)	0.087
Cefalea		1 (5.26%)	1 (11.11%)	0 (0%)	0.474
Fever or Dyspnea		17 (89.47%)	8 (88.89%)	9 (90%)	1.000
Cough or Dyspnea		16 (84.21%)	9 (100%)	7 (70%)	0.211
Cough or Odynophagia		12 (63.15%)	5 (55.56%)	7 (70%)	0.649
**Comorbidities**					
Diabetes		7 (36.84%)	2 (22.22%)	5 (50%)	0.349
Hypertension		9(47.36%)	5 (55.56%)	5 (50%)	1.000
Respiratory diseases		9(47.36%)	2 (22.22%)	5 (50%)	0.349
Cardiac diseases		4 (21.05%)	2 (22.22%)	2 (20%)	1.000
Neurological diseases		1(5.26%)	1 (11.11%)	0 (0%)	0.473
Diabetes or respiratory dis.		12 (63.15%)	3 (33.33%)	9 (90%)	**0.019**
Diabetes or hypertension		11 (57.89%)	5 (55.56%)	6 (60%)	1.000
Respiratory or hypertension		15 (78.95%)	6 (66.67%)	9 (90%)	0.303
Hospitalization		12 (63.15%)	3 (33.33%)	9 (90%)	**0.019**

References: ^1^, Fisher’s exact test except when indicated; ^2^, Kruskal Wallis test. Values between brackets correspond to the percentages refered to the n value indicated on the head of each column

Chronic medical disorders were present in 73% of COVID-19 patients, with hypertension being the most common comorbidity, followed by diabetes, respiratory, cardiac, and neurological diseases. Diabetes was the most frequent illness among non-survivors (Table 2). When the patients were grouped by the presence of diabetes or respiratory diseases, the difference was significatively higher in non-survivors (*p* = 0.019). Related to the symptoms found in these patients, dyspnea was most common with non-survivors and cough with survivors, while the prevalence of fever was similar in both groups (Table 2).

### Genome sequencing, lineage classification and phylogenetic analysis of the Cordoba SARS-CoV-2 strains

The corresponding genome sequences (*n* = 19) were 29,715 to 29,754 nucleotides-long, covered the whole coding region in more than 99% of the genomes, and were submitted to the NCBI Virus database [9]. SARS-CoV-2 lineage assignments were performed using the Phylogenetic Assignment of Named Global Outbreak LINeages nomenclature (Pangolin) COVID-19 Lineage Assigner [10–12] (https://pangolin.cog-uk.io/;https://cov-lineages.org). Five B.1-like lineages were identified, the most prevalent were B.1.499 (9 genomes) and B.1.1.33.3 (also known as N.3, 7 genomes). We also found strains that belong to lineages with less circulation frequency in Argentina, including B.1, B.1.1.1, and B.1.1.33 (Table 1). We found no significant differences between lineage found in survivors and non-survivors.

**Fig. 1:**
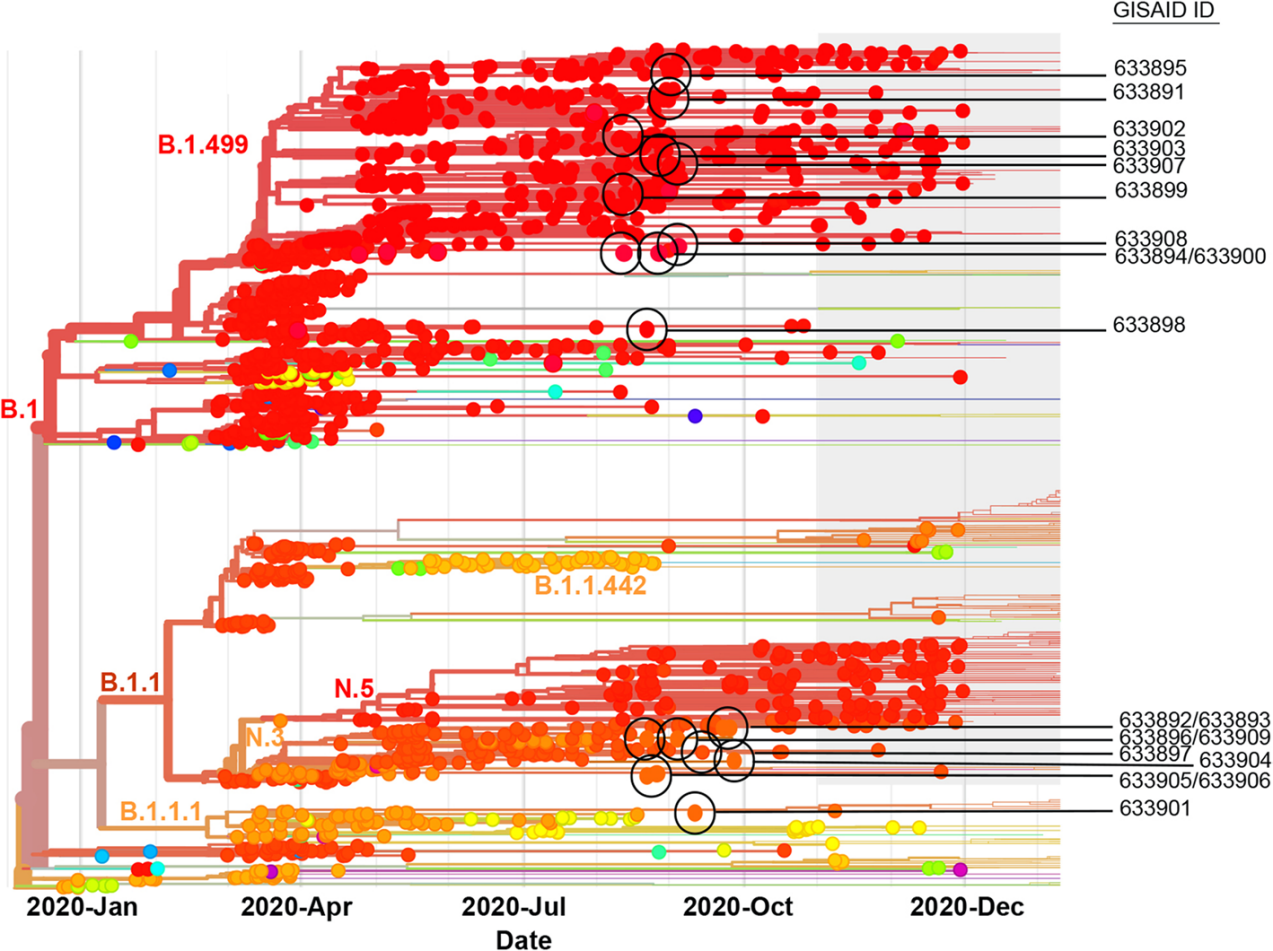
Phylogenetic analysis of the SARS-CoV-2 genomes. This graphic shows the time-resolved phylogeny of the 19 SARS-CoV-2 genomes analyzed in this study combined with the other 1129 genomes (from the GISAID EPICoV database) sampled in Argentina between January 2020 and December 2020. Our strains are indicated with black circles/lines, and the strain names are mentioned in the column on the right. The length of the branches represents the distance in time. The color codes represent the different lineages

Phylogenetic analyses were performed against a background of 1129 SARS-CoV-2 sequences from Argentina in January–December 2020 (GISAID EpiCoV database, [13], https://www.gisaid.org) and analyzed with NextClade V1.6.0 [14]. The hCov-19/Wuhan/WIV04/2019 strain was used as a reference. Time-resolved phylogenetic analysis confirmed that SARS-CoV-2 sequences were grouped into two major lineages, B.1.499 and N.3, which showed higher diversity than B.1, B.1.1, B.1.1.1, B.1.1.442, and N.5 (Fig. 1).

### Analysis of mutations in the SARS-CoV-2 genomes

**Fig. 2:**
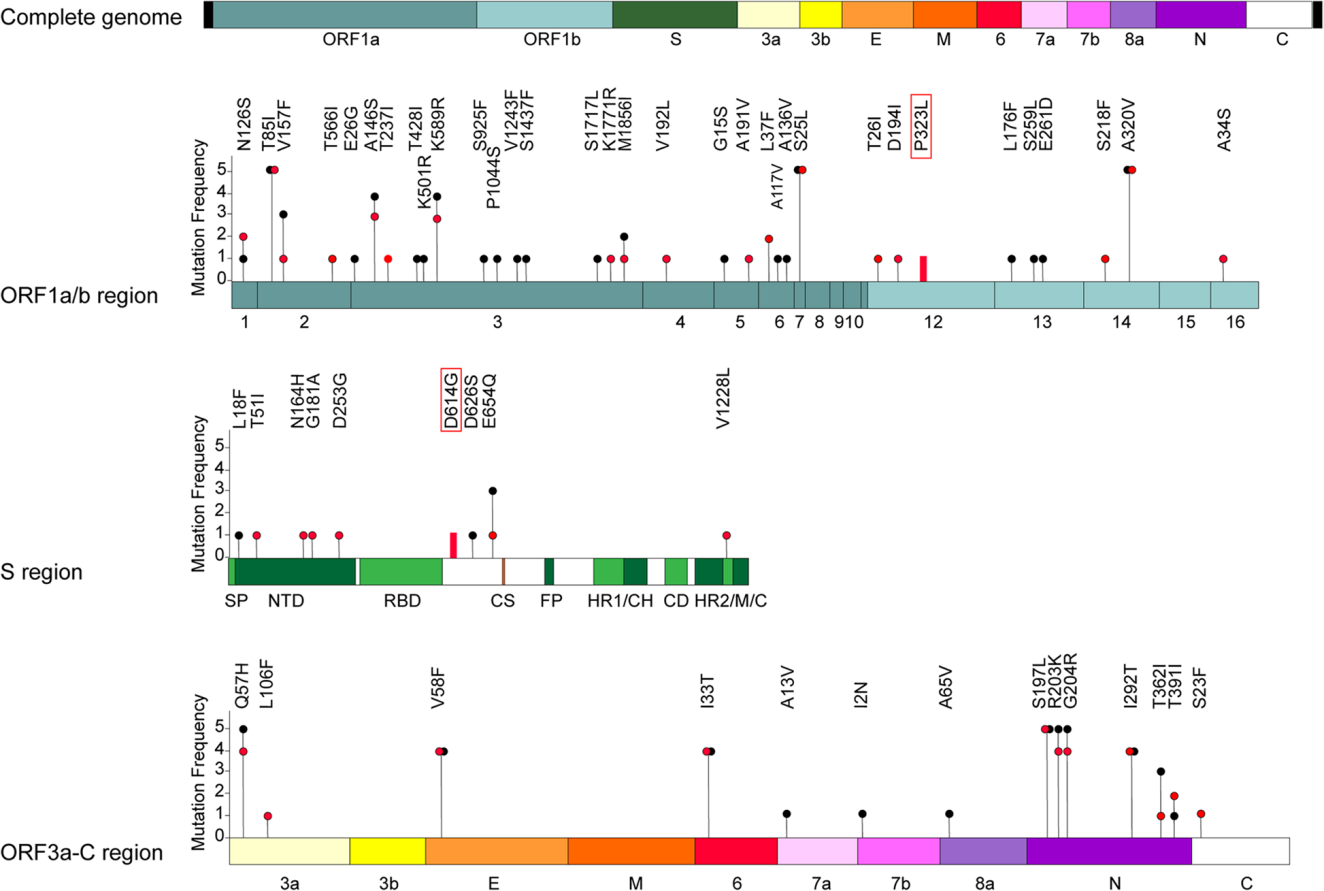
Distribution of missense mutations along the SARS-CoV-2 genome. Schematic representation showing the distribution of missense mutations found in genomes of SARS-CoV-2 obtained from COVID-19 patients. Amino acid mutations are shown by vertical lines in different genome regions (ORF1a/b, S, and ORF3a-C). Mutations identified in survivors are indicated with red circles, and mutations found in non-survivors are indicated with black circles. The abbreviations of genes modified and respective amino acid changes are indicated above the nucleotide changes

Mutations in the SARS-CoV-2 genome sequences were identified using CoVsurver [13] with hCov-19/Wuhan/WIV04/2019 as the reference strain. All 19 genomes presented 56 distinct missense mutations (Table S1, Fig. 2), with D614G (S: Surface glycoprotein) and P323L (RdRp; RNA dependent RNA polymerase) present in all of them (Fig. 2). The Nsp3 (*n* = 13), S (*n* = 9) and N (*n* = 6) proteins have a greater diversity of mutations (Fig. 2) than the rest of the ORFs.

**Fig. 3:**
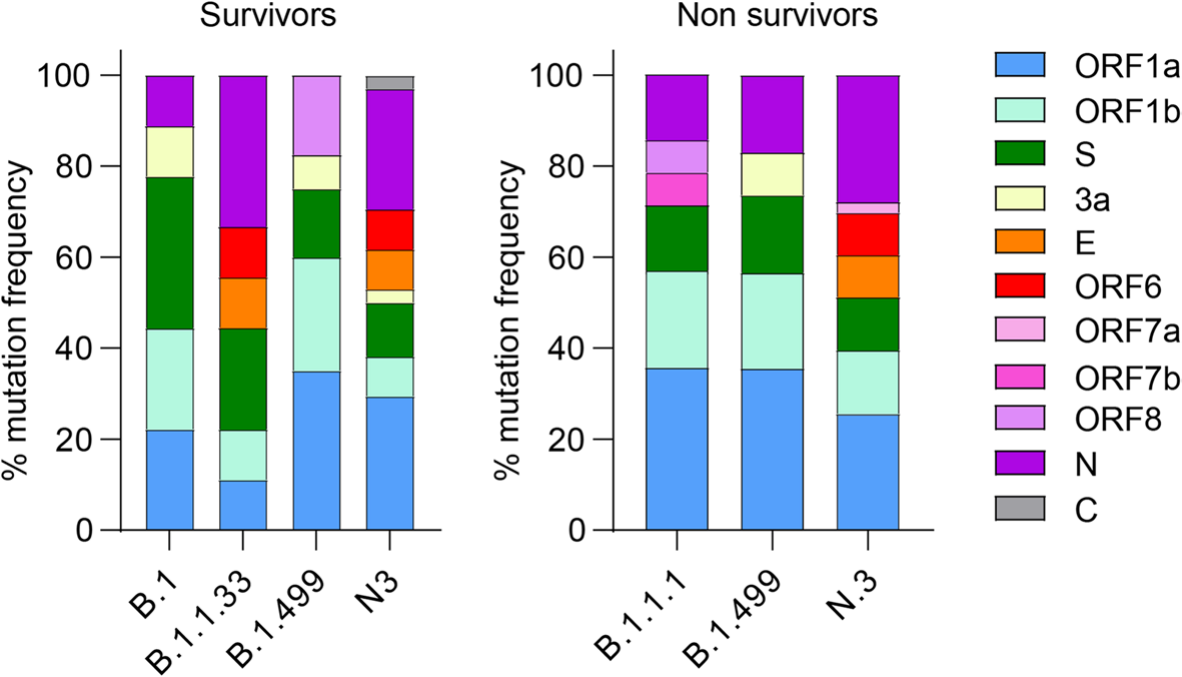
SARS-CoV-2 mutation frequency in different lineages. Percentage distribution of mutations along different SARS-CoV-2 genes are indicated by color codes. Columns show the mutation frequency for each lineage, mutations found in survivors are shown on the left panel, and mutations identified in non-survivors on the right panel. Nsp12:P323L and S:D614G were found in all genomes and are indicated with thick red lines

In genomes from non-survivors, there was a significant predominance of missense mutations in non-structural proteins (*p* = 0.038) (Fig. 3, Table S1). Eight of the 13 different mutations identified in Nsp3 were found in genomes from non-survivors (*p* = 0.017) (Table S1).

The D614G mutation in Spike, a protein that interacts with the human ACE2 receptor, is pivotal for viral entry into the host cells [15] and is linked to enhanced viral transmission [15, 16], was found in all genomes, as previously noted. D614G was the only mutation found in the Spike protein in N.3 lineage strains, but additional S mutations were found in other lineages (Table 1, Fig. 2).

**Fig. 4:**
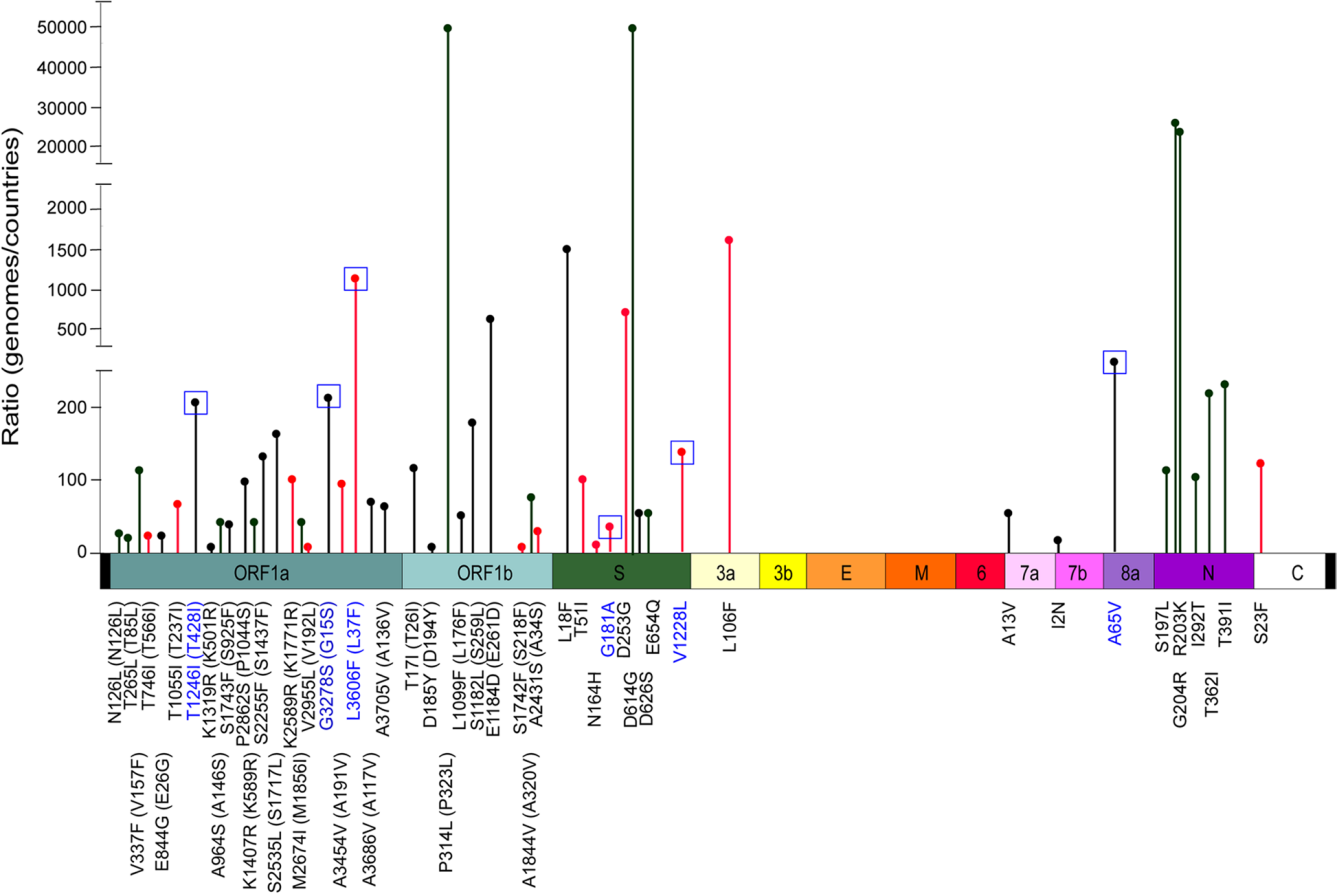
Prevalence of mutations found in this study during evolution of SARS-CoV-2. Schematic representation showing the prevalence of mutations found in genomes of SARS-CoV-2 obtained from COVID-19 patients. In the *y* axis is indicated a ratio used as a spreading indicator, which was estimated using the number of SARS-CoV-2 genomes in which each mutation was found, and the number of countries where these mutations were reported. Amino acid mutations are shown by vertical lines in different genome regions. Mutations identified in survivors are indicated with red circles, in non-survivors with black circles, and in both groups in green. The abbreviations of genes modified and respective amino acid changes are indicated above the nucleotide changes

Twenty-one specific mutations were only detected in the genomes of non-survivors, while 14 were only found in the genomes of survivors (Fig. 2, Fig. S1). To analyze the prevalence of these mutations during the SARS-CoV-2 evolution, each mutation was analyzed by the Lineage/Mutation Tracker [17], enabled by data from GISAID [13], which allows the access to a database with 10,627,993 genome sequences of SARS-CoV-2 (on May 28th, 2022). For these analyses, we used the number of SARS-CoV-2 genomes in which each mutation was found, the number of countries where these mutations were reported, and we obtained a rate value (No. genomes/No. countries) that we used as a spreading indicator (Fig. 4). All of these mutations emerged in the first semester of 2020, and they presented different grades of prevalence (Fig. 4, Table S2). Importantly, they were conserved throughout the evolution of SARS-CoV-2 and are still being detected today (Table S2). Argentina was one of the countries with a major prevalence of the T566I (Orf1a-Nsp2), E26G, T428I (Orf1a-Nsp3), G15S (Rrf1a-Nsp5), D194Y (Orf1b-Nsp12), A34S (Orf1b-Nsp16) mutations. In this sense, most of the S mutations (L18F, T51I, N164H, G181A, D253G, A626S) also showed this spreading capacity in our country (Fig. 4, Table S2).

To better predict the functional effect of these mutations and to investigate whether the presence of mutations in SARS-CoV-2 was associated with COVID-19 patient survivorship, the genomes were analyzed using the Provean V1.1 software [18]. We found 14 mutations in the SARS-CoV-2 genomes predictive of reduced virus fitness (herein referred to as deleterious mutations), which were distributed in ORFs encoding the Leader (1/1), Nsp2 (2/3), Nsp3 (1/13), Nsp7 (1/1), Nsp12 (1/3), Nsp13 (1/3), Nsp14 (1/2), Orf3a (2/2), E (1/1), Orf6 (1/1) and Orf10 (1/1) (Table S1). However, most mutations (43/56) were predicted as neutral. There was no link found between viral deleterious mutations, specific ORF mutations, and survivorship.

We also analyzed the impact of codon bias in the SARS-CoV-2 genomes, and the most abundant mutations were C > U (48.2%), G > U (19.7%), A > G (12.5%), G > C (7.1%), and G > A (5.3%). Of the 56 missense mutations detected, 40 (71.4%) and 16 (28.6%) involved transitions and transversions, dominated by C > U and G > U conversions, respectively. In general, the incidence of transitions was predominant (81.2%) in genes encoding non-structural proteins (*p* = 0.036) (Table S1).

## Discussion/conclusions

The goal of this research was to identify the SARS-CoV-2 lineages that were circulating in the first wave of the COVID-19 pandemic in the Province of Cordoba, Argentina. We identified five B.1-derived lineages; with the most common being N.3. This is consistent with N.3 being the predominant SARS-CoV-2 lineage in Argentina and identified in Paraguay, Chile, Peru, Mexico, and the United States (GISAID virus repository, https://www.gisaid.org). We also detected other lineages such as B.1, which originated from the Northern Italian outbreak at the start of 2020 [19] and produced the first SARS-CoV-2 outbreak in Cordoba in April 2020; B.1.1.1, a lineage that originated in England and spread primarily in Europe and Peru; and B.1.1.33, a lineage that originated in Brazil and was associated with one of the first SARS-CoV-2 outbreaks in Brazil in April 2020 [20]. Time-resolved phylogenetic analysis revealed that the 19 SARS-CoV-2 sequences in this report belonged to two major lineages, B.1.499 and N.3, and were derived from previously identified strains circulating in Argentina. Both lineages displayed significant genomic variability, with B.1.499 exhibiting greater diversity than N.3 during the start of the COVID-19 pandemic in Argentina in the first semester of 2020.

The evolution of SARS-CoV-2 has led to a higher incidence of mutations in regions corresponding to ORF1ab, Spike, N, and ORF8 compared to E, M, ORF6, ORF7a, and ORF7b [21]. We also found a high frequency of variants in Spike, N, ORF1ab, and NSP3, as previously described [22], indicating that these genes are more susceptible to genetic variations.

In comparison with the reference genome, we identified 56 mutations, of which 43 were neutral and 13 were considered deleterious and mostly contained in the *orf1ab* gene. These results are consistent with previous reports [23], suggesting that most variations in the structural proteins of SARS-CoV-2 are neutral despite amino acid changes, although few deleterious mutations have been found in the functional domains of the S (RBD, FP, HR1, and HR2) and N (CTD and NTD) proteins.

In this work, we found known S mutations, such as L18F (linked to NTD-binding antibody escape) [15, 24], T51I, G181A [25], D253G, A626S (a destabilizing S mutation) [16], E654 [25], and V1228L [23]. The N164H mutation was found in only one genome, in the NTD region of the Spike protein. Recently, S:L18F was found in genomes sequences that belong to the Alpha, Beta and Gamma variants, and obtained from COVID-19 patients in South America, USA and India [26].

A previous study indicated that deceased patients have more deleterious than neutral mutations/variants when compared to asymptomatic patients [22]. Mutations such as T428I (*nsp3/orf1ab*), G15S (*nsp5/orf1ab*), and A65V (*orf8*) (Table S1), which were identified in SARS-CoV-2 samples from non-survivors of COVID-19 by Laskar & Ali [22], were also identified in non-survivor patients in our sample set. Likewise, mutations such as L37F (*nsp6*), S:G181A, and S:V1228L, which were identified in SARS-CoV-2 samples from survivors of COVID-19 in the mentioned study [22], were also identified by us in samples corresponding to survivors.

In another work, certain SARS-CoV-2 mutations were associated with the clinical outcome of COVID-19 patients from India. Two mutations (S:D614G and Nsp14:P323L), which were found in all the genomes analyzed in our study, as well as Orf3a:Q57H and N:R203K, also found in some genomes described here, showed a higher incidence in non-survivors [27]. The S:D614G, Nsp14:P323L and N:R203K mutations, in addition to N:G204R, were the most frequent ones during the 5 waves of pandemic in Iran. These authors also reported the presence of other mutations in common with our work, such as Nsp3:S1717L, Nsp6:L37F, Nsp13:L176F, Nsp13:S259L and N:Q57H. It has been described that the N:Q57H and N:R203K/G204R substitutions produce changes in the structure of proteins, which alter the binding affinity of intraviral protein-protein interactions during assembly and release of coronavirus It has been proposed that these changes might be associated with virus evolution and beneficial for the viral pathogenesis [28].

Related to the evolution of the Gamma (P.1) lineage, which had a high incidence in South America, it has been reported in SARS-CoV-2 samples from the State of Amazonas (Brazil) the presence of mutations such as Nsp12:P323L, S:18F, S:D614G and N:R203K/G204R [29]. These mutations were coincident with those found in our study, which were isolated before to the spread of the Gamma variant, suggesting that they could be part of the evolution of this lineage in our region.

All mutations described here showed different grades of prevalence, and are being detected in different countries at present. Mutations such as Nsp2:T566I, Nsp3:E26G, Nsp3:T428I, Nsp5:G15S, Nsp12:D194Y, Nsp16:A34S, as well as those found in the Spike protein (L18F, T51I, N164H, G181A, D253G, A626S) displayed a higher predominance in Argentina. These results suggest that these mutations play a role in the evolution of different lineages where they were identified.

In general, the studied COVID-19 patients displayed common symptoms and comorbidities as previously described [30]. The non-survivors showed a tendency to be male and older, consistent with earlier findings [30–32]. In particular the group aged 76 to 85 years was significantly enriched compared to survivors. Patients with a history of diabetes or respiratory diseases, as well as those patients with a clinical status that required hospitalization, were associated with non-survivors, as reported [30].

In conclusion, this work displays a comparative landscape of mutations corresponding to a cohort of samples obtained for survivors and non-survivors COVID-19 patients, with a predominance of missense mutations in non-structural proteins and Nsp3 mutations in non-survivors. We found that certain factors, such as hospitalization, age and diabetes or respiratory diseases, are relevant in determining clinical outcomes of these patients. Clearly, this genomic analysis is descriptive, and the specific mutations related to survivors and non survivors do not necessarily correlate with the severity of clinical illness. However, our results are in part coincident with those obtained by Laskar & Ali [22] and Maurya et al. [27], as mentioned. We found that they are spread with different grades of prevalence, and we propose that these mutations should be considered in studies of pathogenesis and evolution of SARS-CoV-2. Further analyses beyond the scope of this report are warranted. Altogether, our study provides additional genomic data to better understand the evolution of the SARS-CoV-2 variants that spread in the Central Region of Argentina during the first wave of the COVID-19 pandemic.

## Methods

### Sample collection

Nasopharyngeal swab samples were collected from suspected COVID-19 patients in multiple sites in the Province of Cordoba, Argentina (Table 1) in September 2020. Samples were placed in Viral Transport Medium (GIBCO) and transported to the Central Laboratory. RNA purification was performed using the MagaBio plus Virus RNA Purification Kit II (BioFlux) and using the GenePure Pro Nucleic Acid Purification System NPA-32P (Bioer). RNA samples were tested before 8 h for SARS-COV-2 by qPCR according to the protocol described by DisCoVery SARS-CoV-2 RT-PCR Detection Kit (Safecare Biotech Hangzhou Co., Ltd., China). From the total of confirmed COVID-19 cases, we randomly selected 9 survivors and 10 non-survivor patients. We used a stratified random sampling procedure, we divided the patient population into two groups, survivors and non-survivors, and in each group, we randomly select patients using Research Randomizer software (https://www.randomizer.org) [33]. The corresponding medical records were reviewed to compile epidemiological metadata.

### Viral sequencing

SARS-CoV-2 sequencing was performed as described previously [34]. Briefly, total RNA from nasopharyngeal swab specimens was subjected to complementary DNA (cDNA) synthesis with random hexamers using ProtoScript II (New England Biolabs, E6560), followed by whole-genome amplification with custom-designed tiling primers and library preparation with the Nextera XT DNA Sample Preparation Kit (Illumina, FC-131-1096). The Illumina MiSeq platform was used to sequence Nextera XT libraries in a paired-end 2 × 150 nt run format.

### Sequence data analysis

Illumina SARS-CoV-2 read sequences were assembled into complete genomes using a custom reference-based (MN908947.3) pipeline, https://github.com/mjsull/COVID_pipe [35].

### Phylogenetic, spatio-dynamic and mutation prevalence analysis

To generate a phylogenetic and divergence tree, we downloaded 1129 SARS-CoV-2 genome sequences originating from Argentina during January–December 2020 from the GISAID EpiCoV database [13] (https://www.gisaid.org).

Multiple sequence alignment was performed using Multiple Sequence Comparison by Log- Expectation (MUSCLE) software implemented in Molecular Evolutionary Genetics Analysis software (MEGA) version 10.2.6 [36].

The sequences were analyzed using NextStrain tools (https://nextstrain.org), such as NextClade V1.6.0 [14], and classified by Pangolin lineages. Mutations were identified using the GISAID CoVSurver (www.gisaid.org/epiflu-applications/covsurver-mutations-app) [13]. The hCov-19/Wuhan/WIV04/2019 strain was used as a reference (Accession number NC-045512.2).

The prevalence of the SARS-CoV-2 mutations was analyzed by Lineage/Mutation Tracker, available at https://outbreak.info/situation-reports [17], using the database with 10,627,993 genome sequences from GISAID [13].

### Calculating predicted effect of variants in PROVEAN

The amino acid sequences of each SARS-CoV-2 protein analyzed in this study were uploaded to PROVEAN (Protein Variation Effect Analyzer) (http://provean.jcvi.org/index.php) [18, 37]. Every variant observed in the mutated proteins was compared against the reference sequence (EPI_ISL_402124; WIV04; Wuhan) [38]. Each variant was either predicted to be ‘deleterious’ or ‘neutral’.

### Statistical analysis

Statistical analysis was performed using R software [39] (www.R-project.org). The continue variable age was separated into five different classes. Each class was transformed into a binary categorical variable (belonging to the class) and was evaluated separately. Categorical variables were expressed as counts and continuous variables as the median. A nonparametric Fisher exact test was performed to assess the association between survival/non-survival and categorical variables, and the *p* values were obtained from 2-sided tests using 0.05 as the significance level. The Kruskal-Wallis test was used for association with continuous variables.

## Supplementary Information

**Additional file 1: Figure S1.**

**Additional file 2: Table S1.**

**Additional file 3:** **Table S2.**

**Additional file 4: Table S3.**

## Data Availability

All relevant data are within the paper and its Additional Information files. The 19 SARS-CoV-2 strains sequences obtained in this study were submitted to the NCBI Virus database and the accession numbers are the following MW633891.1–633909.1. The corresponding information about strains is resumed in Table 1.

## Abbreviations

COVID: Corona Virus Disease
GISAID: Global Initiative on Sharing Avian Influenza Data
MEGA: Molecular Evolutionary Genetics Analysis software
MUSCLE: Multiple Sequence Comparison by Log- Expectation
SARS-CoV-2: severe acute respiratory syndrome coronavirus 2
