# Supplementary figures and images for: Phylogenetic analysis and comparative genomics of SARS-CoV-2 from survivor and non-survivor COVID-19 patients in Cordoba, Argentina
Source: BMC Genomics. 2022 Jul 14;23:510. doi: 10.1186/s12864-022-08756-6 (PMC9282626; doi:10.1186/s12864-022-08756-6)

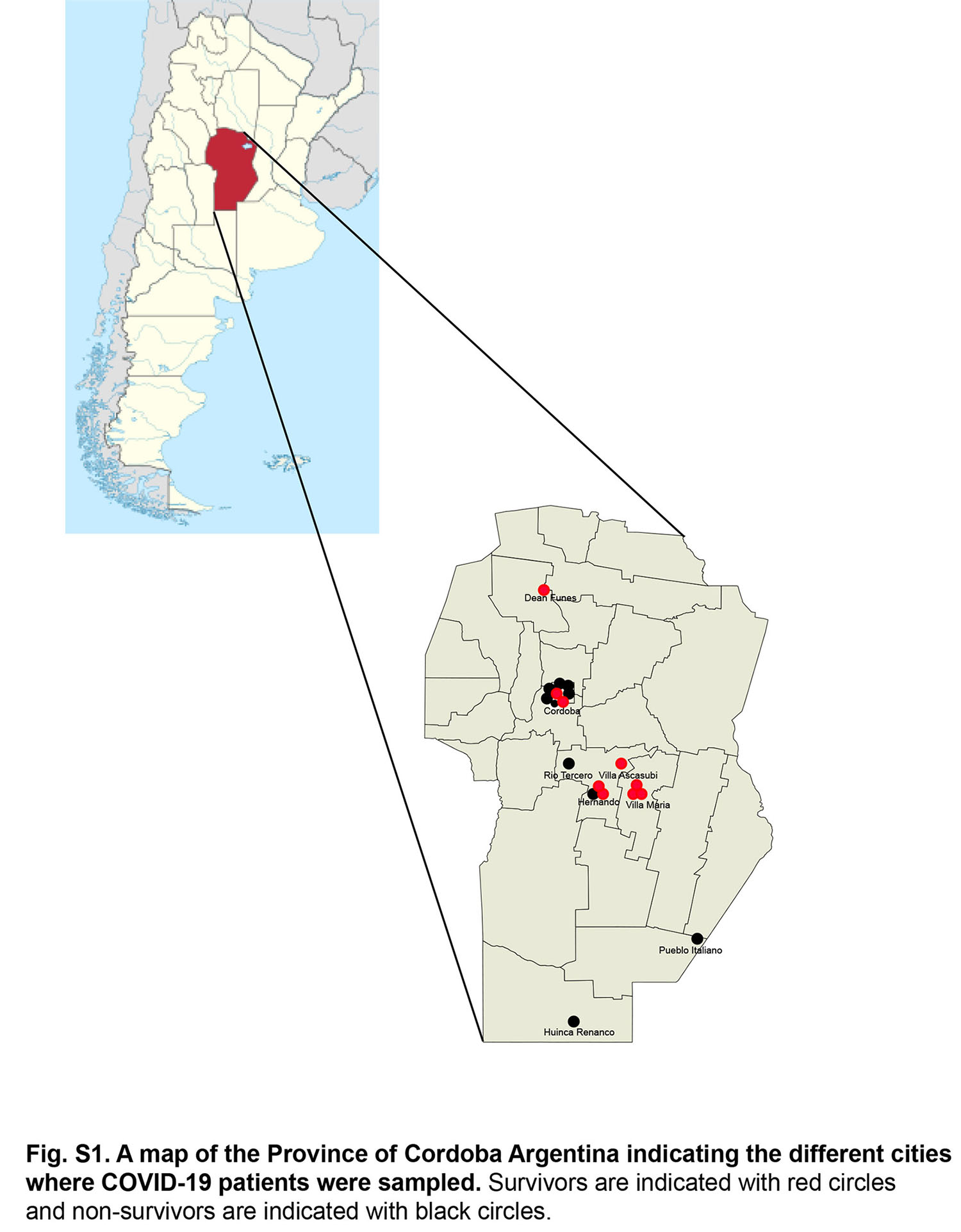

Supplement: Supplementary file 1 — Additional file 1: Figure S1. [file 12864_2022_8756_MOESM1_ESM.jpg]
