# Supplementary material for: Phylogenetic analysis and comparative genomics of SARS-CoV-2 from survivor and non-survivor COVID-19 patients in Cordoba, Argentina
Source: BMC Genomics. 2022 Jul 14;23:510. doi: 10.1186/s12864-022-08756-6 (PMC9282626; doi:10.1186/s12864-022-08756-6)
Supplement: Supplementary file 2 — Additional file 2: Table S1. [file 12864_2022_8756_MOESM2_ESM.pdf]

Table S1. Distribution of neutral and deleterious mutations along with different proteins of SARS-CoV-2 isolated from survivors and non-survivors patients.

Viral Protein	Variant	Mutation type	PROVEAN score[#]	Prediction (cutoff= -2.5)[#]	Specific for non-survivors	Specific for survivors	Presence in both (NS/S)
**Leader**							
	N126S	A>G TS	-3.800	Deleterious			+
**Nsp2**							
	T85I	C>T TS	-4.090	Deleterious			+*
	V157F	G>T TV	-1.333	Neutral			+
	T566I	C>T TS	-3.222	Deleterious	+		
**Nsp3**							
	E26G	A>G TS	-3.260	Deleterious	+		
	A146S	G>T TV	-0.360	Neutral			+
	T237I	C>T TS	-0.263	Neutral		+	
	T428I	C>T TS	-0.527	Neutral	+*		
	K501R	A>G TS	-1.254	Neutral	+		
	K589R	A>G TS	-1.004	Neutral			+
	S925F	C>T TS	-1.857	Neutral	+		
	P1044S	C>T TS	-0.446	Neutral	+		
	V1243F	G>T TV	-0.697	Neutral	+		
	S1437F	C>T TS	-0.521	Neutral	+		
	S1717L	C>T TS	-0.602	Neutral	+		
	K1771R	A>G TS	-0.857	Neutral		+	
	M1856I	G>A TS	0.463	Neutral			+
**Nsp4**							
	V192L	G>C TV	-0.282	Neutral		+	
**Nsp5**							
	G15S	G>A TS	0.056	Neutral	+*		
	A191V	C>T TS	1.133	Neutral		+	
**Nsp6**							
	L37F	G>T TV	-1.369	Neutral		+*	
	A117V	C>T TS	0.312	Neutral	+		
	A136V	C>T TS	-0.906	Neutral	+		
**Nsp7**							
	S25L	C>T TS	-4.272	Deleterious			+*
**Nsp12**							
**(RdRp)**	T26I	C>T TS	-1.867	Neutral	+		
	D194Y	G>T TS	-7.097	Deleterious	+		
	P323L	C>T TS	-0.865	Neutral			+*
**Nsp13**							
**(helicase)**	L176F	C>T TS	-3.665	Deleterious	+		
	S259L	C>T TS	-2.775	Deleterious	+		
	E261D	G>T TV	-0.243	Neutral	+		
**Nsp14**							
	S218F	C>T TS	-4.095	Deleterious		+	

	A320V	C>T TS	-1.694	Neutral			+
**Nsp16**							
	A34S	G>T TV	-1.383	Neutral		+	
**Spike**	L18F	C>T TS	0.781	Neutral	+		
	T51I	C>T TS	-1.219	Neutral		+	
	N164H	A>C TV	-0.360	Neutral		+	
	G181A	G>C TV	0.396	Neutral		+*	
	D253G	A>G TS	-0.440	Neutral		+	
	D614G	A>G TS	0.598	Neutral			+*
	A626S	G>T TV	0.523	Neutral	+		
	E654Q	G>C TV	-0.177	Neutral			+
	V1228L	G>T TV	-0.457	Neutral		+*	
**Orf3a**	Q57H	G>T TV	-3.286	Deleterious			+*
	L106F	C>T TS	-3.629	Deleterious		+	
**E**							
	V58F	G>T TV	-4.000	Deleterious			+
**Orf6**							
	I33T	T>C TS	-4.714	Deleterious			+*
**Orf7a**							
	A13V	C>T TS	-1.825	Neutral	+		
**Orf7b**	I2N	T>A TV		Neutral	+		
**Orf8**							
	A65V	C>T TS	1.222	Neutral	+*		
**N**							
	S197L	C>T TS	-2.221	Neutral			+
	R203K	G>A TS	-1.604	Neutral			+*
	G204R	G>C TV	-1.656	Neutral			+*
	I292T	T>C TS	-2.017	Neutral			+*
	T362I	C>T TS	-1.722	Neutral			+
	T391I	C>T TS	-0.611	Neutral			+
**Orf10**							
	S23F	C>T TS	-6.000	Deleterious		+	

References: TS: transition; TV: transversion; *, residues described by Laskar & Ali (2021). +, presence o mutations; N; non-survivors; S: survivors.
