# Supplementary material for: Phylogenetic analysis and comparative genomics of SARS-CoV-2 from survivor and non-survivor COVID-19 patients in Cordoba, Argentina
Source: BMC Genomics. 2022 Jul 14;23:510. doi: 10.1186/s12864-022-08756-6 (PMC9282626; doi:10.1186/s12864-022-08756-6)
Supplement: Supplementary file 3 — Additional file 3: Table S2. [file 12864_2022_8756_MOESM3_ESM.pdf]

Table S2. Data about prevalence of mutations during evolution of SARS-CoV-2 worlwide.

Protein	Mutations[1]	Clinical outcome[2]	Number of genomes[3]	Number of countries[4]	Ratio #genomes/#countries[5]	Predominant Pango Lineages[6]	Cummulative prevalence[7]	Firts report[8]	Last report[9]
**Orf1a (Nsp1)**	N126S (N126S)	NS/S	1,184	40 (Zam; Swe; Leb)	29,6	B.1.1.277 (19); B.1.302(1	<0,5%	March 12, 2020	May 22, 2022
**Orf1a (Nsp2)**	T265L (T85L)	NS/S	13	1 (US)	13	ND	<0,5%	March 24, 2021	May 24, 2021
	V337F (V157F)	NS/S	10,411	90 (Mon; Tun; Arg)	115,7	C.20 (15); AY.80 (8)	<0,5%	March 15, 2021	May 20, 2021
	T746I (T566I)	S	477	40 (Ukr; Egy, Arg)	11,9	multiple	<0,5%	April 8, 2020	May 3, 2022
**Orf1a (Nsp3)**	E844G (E26G)	NS	330	25 (Ang, Ghana, Arg)	13,1	multiple	<0,5%	July 26, 2020	May 3, 2022
	A964S (A146S)	NS/S	878	24 (Arg; Ven; Bul)	36,6	N.3 (17)	<0,5%	June 19, 2020	May 6, 2022
	T1055I (T237I)	S	6,292	88 (Guy, Rom; Qat)	71,5	D.5 (99)	<0,5%	March 7, 2020	May 7, 2022
	T1246I (T428I)	NS	27,982	130 (Egy; Peru, Arg)	215,2	multiple (C.36; C.37)	<0,5%	March 2, 2020	May 7, 2022
	K1319R (K501R)	NS	184	28 Cam; Rus; Peru)	6,6	multiple	<0,5%	Oct 21, 2020	April 25, 2022
	S1743F (S925F)	NS	740	42 (Gui, Arg; Col)	17,6	multiple	<0,5%	June 14, 2020	April 25, 2022
	P1862S (P1044S)	NS	8,712	90 (Guy; Est; Ire)	96,8	B.1.232 (80)	<0,5%	March 12, 2020	May 5, 2022
	K1407R (K589R)	NS/S	1,026	33 (Arm; Arg; Par)	31,1	N.3 (18); B.1.637 (5)	<0,5%	March 28, 2020	May 11, 2022
	S2255F (S1437F)	NS	8,927	70 (Alb; Gree; Swi)	127,5	B.1.641 (100)AY.92 (37)	<0,5%	March 22, 2020	May 7, 2022
	S2535L (S1717L)	NS	17,645	107 (Hon; Leb; Gua)	164,9	B.1.627 (97%)	<0,5%	March 9, 2020	May 9, 2022
	K2589R (K1771R)	S	7,137	104 (Lyb; Nig; Kaz)	68,6	B.1.177.26 (79%)	<0,5%	Febr. 3, 2020	May 5, 2022
	M2674I (M1856I)	NS/S	1,783	65 (Nam; Ang; Arg)	27,4	B.1.499 (4); C.16 (3)	<0,5%	Apri 4, 2020	May 4, 2022
**Orf1a (Nsp4)**	V2955L (V192L)	S	173	21 (Bol; Ita; Mex)	8,2	multiple	<0,5%	April 25, 2020	April 12, 2022
**Orf1a (Nsp5)**	G3278S (G15S)	NS	27,004	125 (Egy; Peru, Arg)	216,1	multiple (C,37)	<0,5%	March 2, 2020	May 4, 2022
	A3454V (A191V)	S	8,994	95 (Nic; Mal: Tun)	94,7	B.1.177.20(100)/B.1.1.93(100)	<0,5%	Febr. 21, 2020	May 5, 2022
**Orf1a (Nsp6)**	L3606F (L37F)	S	207,193	178 (Jor; Ice: Par)	1164,3	multiple	2%	Jan. 21, 2020	May 6, 2022
	A3686V (A117V)	NS	2,821	59 (Mac; Lat;Nic)	48,6	B.1.617.3 (89%); C,30 (7%)	<0,5%	March 11, 2020	May 5, 2022
	A3705V (A136V)	NS	7,237	88 (Mon; Zam; Col)	82,3	B.1.426B.1.177.43 (49% c/u)	<0,5%	March 10, 2020	May 5, 2022
**Orf1b (Nsp12)**	T17I (T26I)	NS	15,954	130 (Alg; Bel; Cam)	122,7	B.1.614(10); B.1.177.77 (96)	<0,5%	March 8, 2020	May 9, 2022
	D185Y (D194Y)	NS	69	18 (Bel; Nig; Arg)	3,8	multiple	<0,5%	May 25 8, 2020	May 4, 2022
	P314L (P323L)	NS/S	10,577,496	206 (global)	51347,1	multiple	98%	Jan 26, 2020	May 24, 2022

**Orf1b (Nsp13)**	L1099F (L176F)	NS	3,881	69 (Syr; Tai; Ukr)	56,3	B.1.1.483(100); B.1.208(100); D.4(100)	<0,5%	March 2, 2020	May 6, 2022
	S1182L (S259L)	NS	18,356	106 (Tim; Ind; Kos)	173,2	AU.2 (99) AU.3 (98) B.1.466.2(88)	<0,5%	March 15, 2020	May 6, 2022
	E1184D (E261D)	NS	90,521	142 (Hun; Jap; Col)	637,5	B.1.160.8 (100); AB.1(100)	1,00%	Feb 6, 2020	May 4, 2022
**Orf1b (Nsp14)**	S1742F (S218F)	S	295	40 (Pan; Uru; Chile)	7,4	multiple (<1% each)	<0,5%	Apr 12, 2020	May 4, 2022
	A1844V (A320V)	NS/S	4,738	58 (Arg; Sri; Par)	81,7	B.1.498 (100); B.1.499 (99)	<0,5%	March 5, 2020	May 12, 2022
**Orf1b (Nsp16)**	A2431S (A34S)	S	564	28 (Arg; Nep; Ecu)	20,2	B.1.1.518(58); N.2(24)	<0,5%	Jun 15, 2020	Apr 18, 2022
**S**	L18F	NS	245,452	169 (Brazil, Arg, Bol)	1452,4	multiple	2%	Jan. 28, 2020	April 28, 2022
	T51I	S	8,005	71 (Ang; Peru; Ire)	112,8	B.1.177.62 (52%)/B.1.177.61 (39%)	<0,5%	March 18, 2020	April 29, 2022
	N164H	S	255	22 (Arg, Chile, US)	11,6	multiple	<0,5%	Aug 17, 2020	April 19, 2022
	G181A	S	2,366	75 (Lybia, Irak, Arg)	31,6	multiple	<0,5%	March 23, 2020	May 1, 2022
	D253G	S	50,295	98 (Belize, Ecu, US)	513,2	C.19 (100); B.1.526 (100) B.1.426 (98)	<0,5%	Jan 28, 2020	May 5, 2022
	D614G	NS/S	10,627,832	208 (global)	51095,3	multiple	99%	Jan 26, 2020	May24, 2022
	A626S	NS	4,131	70 (Hung, Arg, Nor)	59,3	K.3 (100); B.1.499.1 (100); B.1.1.277 (76)	<0,5%	March 11, 2020	May 13, 2022
	E654Q	NS/S	2,015	63 (Arg; Cyp; Por)	32,1	B.1.562 (67); B.1.36.24 (43); B.1.499 (15)	<0,5%	March 23, 2020	May 5, 2022
	V1228L	S	16,184	116 (Libya, Par, Nig)	139,5	B.1.258 (100); B.1.177 (92); L.3 (92)	<0,5%	Feb 5, 2020	May 3, 2022
**Orf3a**	Q57H	NS/S	561,759	191 (Sau; Hun; Ang)	2941,1	multiple	5%	Jan 26, 2020	May 7, 2022
	L106F	S	239,982	155 (Haiti, Col, Mex)	1548,3	BA.1.15(100); B.1.1.514 (60)	2%	March 10, 2020	May 7, 2022
**Orf7a**	A13V	NS	1,198	48 (Pol; Aus; Spain)	24,5	B.1.1.415(35); B.1.1.524(8)	<0,5%	Feb. 22, 2020	Apr 30, 2022
**Orf7b**	I2N	NS	597	51 (Congo, Iran, Ecu)	11,7	multiple	<0,5%	March 26, 2020	May 2, 2022
**Orf8**	A65V	NS	46,414	136 (Guy, Hun, Aru)	341,3	B.1.1.256(100); B.1.1.240 (97);AJ.1(95)	<0,5%	Feb. 2, 2020	May 10, 2022

**N**	S197L	NS/S	9,707	83 (Nic; Pan; Arg)	116,9	B.1.499 (10); A.2.5.1 (100)	<0,5%	Feb. 4, 2020	May 17, 2022
	R203K	NS/S	5,258,561	203 (Aze; Pal; Uru)	25904,2	multiple	49%	Jan 29, 2020	May 24, 2022
	G204R	NS/S	5,181,167	203 (Iran: Uru; Braz)	25522,9	multiple	48%	Jan 29, 2020	May 24, 2022
	I292T	NS/S	5,793	58 (Uru; Arg; Par)	99,9	N,3 (100); N,4 (100); N,7 (100)	<0,5%	Feb. 15, 2020	May 20, 2022
	T362I	NS/S	28,163	129 (Jam; Mon; Ban)	218,3	B.1.1.451 (100); B.1.36.26 (97)	<0,5%	March 18, 2020	May 23, 2022
	T391I	NS/S	22,076	92 (Leb; Col; Chi)	239,9	B.1.641 (100); B.1.311 (88)	<0,5%	Feb. 19, 2020	May 16, 2022
**Orf10**	S23F	S	15,214	106 (S.Kor; Vie; Kuw)	143,5	B.1.1.123 (92)	<0,5%	Feb. 5, 2020	May 5, 2022

References

[1], Specific mutations found in genome sequences obtained from samples of COVID-19 patients. For Orf1ab mutations, their localization in Nsp are indoicated between brackets.
Mutations described by Laskar et al (2021) are underligned.

[2], Clinical outcomes of the COVID-19 patients are indicated as NS (non-survivors) and S (survivors),

[3], Number of genomes where mutations were found present. GISAID (https://www.gisaid.org) was used as a database, which contains 10,627,993 genomes sequences of SARS-CoV-2 (May 28, 2022),

[4], Number of countries where mutations were found in the SARS-CoV-2 genomes reported in the GISAID database. The most representative countries are mentioned between brackets.
Country names are mentioned with the first three letters.

[5], Ratio (No. genomes/No. countries) as an indicator of mutation spread during the evolution of SARS-CoV-2. This rate was obtained using the number of genomes where the mutations
were detected and the number of countries where mutations were reported.

[6], Predominant Pango lineages corresponding to the genomes that harbor specific mutations. The percentages of the predominance are indicated between brackets.

[7], Apparent cumulative prevalence is the ratio of the sequences containing specific mutations to all sequences collected since the identification of a determined.
mutation worldwide (10,378,682 genome sequences from GISAID).

[8], The date when the mutation was reported for the first time.

[9]; The date when the mutation was reported for the last time.
