# Supplementary material for: Phylogenetic analysis and comparative genomics of SARS-CoV-2 from survivor and non-survivor COVID-19 patients in Cordoba, Argentina
Source: BMC Genomics. 2022 Jul 14;23:510. doi: 10.1186/s12864-022-08756-6 (PMC9282626; doi:10.1186/s12864-022-08756-6)
Supplement: Supplementary file 4 — Additional file 4: Table S3. [file 12864_2022_8756_MOESM4_ESM.pdf]

Table S3. Acknowledgment to the authors of genome sequences deposited in the GISAID database.

Accession ID	Originating Laboratory	Submitting Laboratory	Authors
EPI_ISL_1396412, EPI_ISL_1396413, EPI_ISL_1396414, EPI_ISL_1396415, EPI_ISL_1396416, EPI_ISL_1396417, EPI_ISL_1396418, EPI_ISL_1396419, EPI_ISL_1396420, EPI_ISL_1396421, EPI_ISL_1396422, EPI_ISL_1396423, EPI_ISL_1396424, EPI_ISL_1396426, EPI_ISL_1396427, EPI_ISL_1396429, EPI_ISL_1396430, EPI_ISL_1396431, EPI_ISL_1396432, EPI_ISL_1396433, EPI_ISL_1396434, EPI_ISL_1396435, EPI_ISL_1396436, EPI_ISL_1396437, EPI_ISL_1396438, EPI_ISL_1396439, EPI_ISL_1396440, EPI_ISL_1396441, EPI_ISL_1396442, EPI_ISL_1396443, EPI_ISL_1396444, EPI_ISL_1396445, EPI_ISL_1396446, EPI_ISL_1396447, EPI_ISL_1396448, EPI_ISL_1396449, EPI_ISL_1396450, EPI_ISL_1396451, EPI_ISL_1396452, EPI_ISL_1396453, EPI_ISL_1396454, EPI_ISL_1396455, EPI_ISL_1396456, EPI_ISL_1396457, EPI_ISL_1396458, EPI_ISL_1396459, EPI_ISL_1396460, EPI_ISL_1396461, EPI_ISL_1396462, EPI_ISL_1396463, EPI_ISL_1396464, EPI_ISL_1396465, EPI_ISL_1396466, EPI_ISL_1396467, EPI_ISL_1396468, EPI_ISL_1396469, EPI_ISL_1396470, EPI_ISL_1396471, EPI_ISL_1396472, EPI_ISL_1396473, EPI_ISL_1396474, EPI_ISL_1396475, EPI_ISL_1396476, EPI_ISL_1396477, EPI_ISL_1396478, EPI_ISL_1396479, EPI_ISL_1396480, EPI_ISL_1396481, EPI_ISL_1396482, EPI_ISL_1396483, EPI_ISL_1396484, EPI_ISL_1396485, EPI_ISL_1396486, EPI_ISL_1396487, EPI_ISL_1396488, EPI_ISL_1396489, EPI_ISL_1396490, EPI_ISL_1396491, EPI_ISL_1396492, EPI_ISL_1396493, EPI_ISL_1396494, EPI_ISL_1396495, EPI_ISL_1396496, EPI_ISL_1396497, EPI_ISL_1396498, EPI_ISL_1396499, EPI_ISL_1396500, EPI_ISL_1396501, EPI_ISL_1396502, EPI_ISL_1396503, EPI_ISL_1396504, EPI_ISL_1396505, EPI_ISL_1396506, EPI_ISL_1396507, EPI_ISL_1396508, EPI_ISL_1396509, EPI_ISL_1396510, EPI_ISL_1396511, EPI_ISL_1396512, EPI_ISL_1396513, EPI_ISL_1396514, EPI_ISL_1396515, EPI_ISL_1396516, EPI_ISL_1396517, EPI_ISL_1396518			
see above	Centro de Tecnología en Salud Pública de la Universidad Nacional de Rosario	Laboratorio Mixto de Biotecnología Acuática (LMBA) on behalf of 'Proyecto Argentino Interinstitucional de genomica de SARS-CoV-2' (PAIS Consortium)	Adriana Giri; Agustina Cerri; Ana Cavatorta; Ana Paletta; Diego Chouhy; Elisa Bolatti; Elizabeth Tapia (argenTAG); Federico Remes Lenicov; Flavio Spetale; Gastón Viarengo; Ignacio García Labari; Javier Murillo; Joaquín Ezpeleta; Julian Acosta; Laura Angelone; Leandro Ciappina; María Re; Pablo Casal; Pilar Bulacio; Silvana Spinelli; Silvia Arranz; Sofía Lavista Llanos; Vanina Villanova; Victoria Posner
EPI_ISL_430796, EPI_ISL_430797, EPI_ISL_430798, EPI_ISL_430802, EPI_ISL_430805, EPI_ISL_430806, EPI_ISL_430808, EPI_ISL_792301			
see above	Departamento de Biología y genética molecular, IACA Laboratorios.	Área de Secuenciación del Laboratorio de Virología del Hospital de Niños Dr. Ricardo Gutierrez on behalf of 'Proyecto Argentino Interinstitucional de genomica de SARS-CoV-2' (PAIS Consortium)	A; AS; E; Goya; LE; Lusso; MI; MS; Masciovecchio MV; Mistchenko; Nabaes Jodar; Natale; S; Streitenberger ER; Suárez; Tittarelli; Valinotto; Viegas, M.
EPI_ISL_1394929	Hospital Alemán	Área de Secuenciación del Laboratorio de Virología del Hospital de Niños Dr. Ricardo Gutierrez on behalf of 'Proyecto Argentino Interinstitucional de genomica de SARS-CoV-2' (PAIS Consortium)	Acuña; Alexay; D; Eugenia Ibañez; Goya; LE; Lusso; M; MI; María Paula Della Latta; Nabaes Jodar; Natale; Natalia García Allende; S; Valinotto; Viegas, M.
EPI_ISL_1396364, EPI_ISL_1396365, EPI_ISL_1396366, EPI_ISL_1396367, EPI_ISL_1396368, EPI_ISL_1396369, EPI_ISL_1396370, EPI_ISL_1396371, EPI_ISL_1396372, EPI_ISL_1396373			
see above	Hospital Central Mendoza	Nodo de Secuenciación Tierra del Fuego - Hospital Regional Ushuaia - Centro Austral De Investigaciones Científicas - Universidad Nacional De Tierra Del Fuego on behalf of 'Proyecto Argentino Interinstitucional de genomica de SARS-CoV-2' (PAIS Consortium)	Aguirre Carolina; Alberto Carena; Alejandro Ezequiel Rojas; Andrea Falaschi; Andrea Naser; Bosio Lia; Carlos Espul; Cristina Fernanda Nardi; Fernando Gallego; Héctor Horacio Cuello; Ivan Dario Gramundi; Luciano Lima; Maria Belen Peralta Roca; María Liliana Videla; Pablo Rico; Patricia Robledo; Santiago Guillermo Ceballos; Valeria Fontana
EPI_ISL_1396425, EPI_ISL_1396428	Hospital Español de Rosario	Laboratorio Mixto de Biotecnología Acuática (LMBA) on behalf of 'Proyecto Argentino Interinstitucional de genomica de SARS-CoV-2' (PAIS Consortium)	Adriana Giri; Agustina Cerri; Ana Cavatorta; Ana Paletta; Diego Chouhy; Elisa Bolatti; Elizabeth Tapia (argenTAG); Federico Remes Lenicov; Flavio Spetale; Gastón Viarengo; Ignacio García Labari; Javier Murillo; Joaquín Ezpeleta; Julian Acosta; Laura Angelone; Leandro Ciappina; María Re; Pablo Casal; Pilar Bulacio; Silvana Spinelli; Silvia Arranz; Sofía Lavista Llanos; Vanina Villanova; Victoria Posner
EPI_ISL_476496, EPI_ISL_476561	Hospital Garrahan	Héritas	Andrea Mangano; Cristian Rohr; Dalmacio Pereyra; Fabian Fay; Maria Florencia Fernandez; Martin Vazquez; Mauricio Grisolia; Roberta Crespo
EPI_ISL_1396374, EPI_ISL_1396375, EPI_ISL_1396376, EPI_ISL_1396377, EPI_ISL_1396378, EPI_ISL_1396379, EPI_ISL_1396380, EPI_ISL_1396381, EPI_ISL_1396382			
see above	Hospital Notti Mendoza	Nodo de Secuenciación Tierra del Fuego - Hospital Regional Ushuaia - Centro Austral De Investigaciones Científicas - Universidad Nacional De Tierra Del Fuego on behalf of 'Proyecto Argentino Interinstitucional de genomica de SARS-CoV-2' (PAIS Consortium)	Adriana Recabarren; Aguirre Carolina; Alberto Carena; Alejandro Ezequiel Rojas; Andrea Falaschi; Bosio Lia; Carlos Espul; Clara Pott Godoy; Cristina Fernanda Nardi; Daiana Lang; Fernando Gallego; Ivan Dario Gramundi; Maria Belen Peralta Roca; Noelia Cuglia; Patricia Robledo; Sandra Grucci; Santiago Guillermo Ceballos; Vanina Eibar
EPI_ISL_1395808, EPI_ISL_1395809, EPI_ISL_1395810, EPI_ISL_1395811	Hospital Pediatrico Dr Avelino Castelan, Hospital 4 de Junio Dr Ramon Carrillo	Grupo de Genómica y Bioinformática del Instituto de Investigación de la Cadena Láctea CONICET-INTA on behalf of 'Proyecto Argentino Interinstitucional de genomica de SARS-CoV-2' (PAIS Consortium)	AF; Amadio; Andrea Piedra Buena; Antonieta Cayré; Eberhardt; Gabriela Jurasek; Gili Andrea; Irazoqui; Laura Lescano; Lopez María Cecilia; MF; Marino Goia; Mayra Mendoza
EPI_ISL_476563, EPI_ISL_476565, EPI_ISL_476567, EPI_ISL_476568, EPI_ISL_476571, EPI_ISL_476573, EPI_ISL_615121			
see above	Hospital de Pediatría "Prof. Dr. Juan P Garrahan"	Héritas	Andrea Mangano; Bianca Brun; Cristian Rohr; Dalmacio Pereyra; Fabian Fay; Maria Florencia Fernandez; Martin Vazquez; Mauricio Grisolia; Priscila Aldabe; Roberta Crespo
EPI_ISL_648209, EPI_ISL_648210, EPI_ISL_648211, EPI_ISL_648212, EPI_ISL_648213, EPI_ISL_648214, EPI_ISL_648215, EPI_ISL_648216, EPI_ISL_648218, EPI_ISL_648677			
see above	INBIRS-UBA	Laboratorio Mixto de Biotecnología Acuática (LMBA)	Adriana Giri; Agustina Cerri; Ana Cavatorta; Ana Paletta; Diego Chouhy; Elisa Bolatti; Elizabeth Tapia; Federico Remes Lenicov; Flavio Spetale; Gastón Viarengo; Ignacio García Labari; Javier Murillo; Joaquín Ezpeleta; Julian Acosta; Laura Angelone; Leandro Ciappina; María Re; Pablo Casal; Pilar Bulacio; Silvana Spinelli; Silvia Arranz; Sofía Lavista Llanos; Vanina Villanova; Victoria Posner
EPI_ISL_1395812, EPI_ISL_1395813, EPI_ISL_1395814, EPI_ISL_1395815, EPI_ISL_1395816, EPI_ISL_1395817, EPI_ISL_1395818, EPI_ISL_1395819, EPI_ISL_1395820, EPI_ISL_1395821, EPI_ISL_1395822, EPI_ISL_1395823, EPI_ISL_1395824, EPI_ISL_1395825, EPI_ISL_1395826, EPI_ISL_1395827, EPI_ISL_1395828, EPI_ISL_1395829, EPI_ISL_1395833, EPI_ISL_1395834, EPI_ISL_1395835, EPI_ISL_1395836, EPI_ISL_1395837, EPI_ISL_1395838, EPI_ISL_1395839			
see above	Inmunología del Hospital Perrando e Instituto de Medicina Regional de la UNNE	Grupo de Genómica y Bioinformática del Instituto de Investigación de la Cadena Láctea CONICET-INTA on behalf of 'Proyecto Argentino Interinstitucional de genomica de SARS-CoV-2' (PAIS Consortium)	AF; Amadio; Antonieta Cayré; Eberhardt; Gerardo Deluca; Gustavo Giusiano; Horacio Lucero; Irazoqui; Laura Lescano; MF; Marcelo Marin; María Delia Foussal; María Verónica Gómez; Natalia Andrea Ayala
EPI_ISL_3478829, EPI_ISL_3478830, EPI_ISL_3478832, EPI_ISL_3478836, EPI_ISL_3478837, EPI_ISL_3478838, EPI_ISL_3478839, EPI_ISL_3478840, EPI_ISL_3478841, EPI_ISL_3478842, EPI_ISL_3478843, EPI_ISL_3478844, EPI_ISL_3478845, EPI_ISL_3478846, EPI_ISL_3478847, EPI_ISL_3478848, EPI_ISL_3478849, EPI_ISL_3478850, EPI_ISL_3478851, EPI_ISL_3478852, EPI_ISL_3478853, EPI_ISL_3478854, EPI_ISL_3478857, EPI_ISL_3478858, EPI_ISL_3478859, EPI_ISL_3478861, EPI_ISL_3478863, EPI_ISL_3478866, EPI_ISL_3478867, EPI_ISL_3478868, EPI_ISL_3478869, EPI_ISL_3478870, EPI_ISL_3478871, EPI_ISL_3478873, EPI_ISL_3478874, EPI_ISL_3478875, EPI_ISL_3478876, EPI_ISL_3478877, EPI_ISL_3478878, EPI_ISL_3478879, EPI_ISL_3478880, EPI_ISL_3478881, EPI_ISL_3478882, EPI_ISL_3478883, EPI_ISL_3478884, EPI_ISL_3478886, EPI_ISL_3478893, EPI_ISL_3478894, EPI_ISL_3478895, EPI_ISL_3478897, EPI_ISL_3478898, EPI_ISL_3478899, EPI_ISL_3478900, EPI_ISL_3478901, EPI_ISL_3478902, EPI_ISL_3478903, EPI_ISL_3478904, EPI_ISL_3478905, EPI_ISL_3478906, EPI_ISL_3478907, EPI_ISL_3478908, EPI_ISL_3478910, EPI_ISL_3478911			
see above	Instituto Nacional de Enfermedades Virales Humanas Dr. Julio I. Maiztegui	Laboratorio Mixto de Biotecnología Acuática (LMBA) on behalf of 'Proyecto Argentino Interinstitucional de genomica de SARS-CoV-2' (PAIS Consortium)	Ada Nazar; Adriana Giri; Agustina Cerri; Agustina Pacual; Anabel Sinchi; COFECyT SF-11; Camila Gonzalez; Carina Bonacalza; Carina Sen; Carlos Figueroa; Cintia Fabbri; Diego Chouhy; Elisa Bolatti; Elizabeth Tapia; Flavio Spetale; Florencia Mascali; Focem COF 03/11 COVID-19); Gastón Viarengo; Germán R. Perez; Ignacio García Labari; Javier Murillo; Joaquín Ezpeleta; Julia Brignone; Leandro Ciappina; Maria Laura Casela; Mariana Viegas (Financiamiento: argenTAG; Mariel Feroci; María Alejandra Morales; María Laura Martin; María Re; María de los Angeles Conti; Matías Abalo; Pablo Casal; Pilar Bulacio; Proyecto IP COVID-19 N°08; Silvana Spinelli; Silvia Arranz; Sofía Lavista Llanos; Sylvia Garcia; Vanina Villanova; Victoria Luppo; Victoria Posner
EPI_ISL_792351, EPI_ISL_792352, EPI_ISL_792353	Instituto Nacional de Epidemiología Dr. Jara	Área de Secuenciación del Laboratorio de Virología del Hospital de Niños Dr. Ricardo Gutierrez on behalf of 'Proyecto Argentino Interinstitucional de genomica de SARS-CoV-2' (PAIS Consortium)	CJ; Cimmino; Goya; I; LE; Lusso; MI; MS; Nabaes Jodar; Natale; O; Pagano; S; Uez; Valinotto; Viegas, M.
EPI_ISL_792320, EPI_ISL_792321, EPI_ISL_792322, EPI_ISL_792323, EPI_ISL_792324, EPI_ISL_792325, EPI_ISL_792326, EPI_ISL_792327, EPI_ISL_792328, EPI_ISL_792329, EPI_ISL_792330, EPI_ISL_792331, EPI_ISL_792332			
see above	Instituto de Investigaciones Biomédicas en Retrovirus y SIDA	Área de Secuenciación del Laboratorio de Virología del Hospital de Niños Dr. Ricardo Gutierrez on behalf of 'Proyecto Argentino Interinstitucional de genomica de SARS-CoV-2' (PAIS Consortium)	F; Goya; H; LE; Lusso; MI; MS; Nabaes Jodar; Natale; Remes Lenicov; S; Salomón; Seery; V; Valinotto; Viegas, M.
EPI_ISL_499083	Instituto de Virologia "Dr. J. M. Vanella", Facultad de Ciencias Medicas, Universidad Nacional de Cordoba. Laboratorio Central de la Provincia de Cordoba, Argentina. Ministerio de Salud de la provincia de Cordoba, Argentina.	Laboratorio de Virología, Hospital de Niños Ricardo Gutiérrez, CABA, Argentina.	Adrian Diaz; Brenda Konigheim; Gabriela Barbas; Gonzalo Castro; Javier Aguilar; Lorena Spinsanti; Mariana Viegas.; Mercedes Nabaes; Monica Natale; Sandra Gallego; Sebastian Blanco; Silvina Lusso; Stephanie Goya
EPI_ISL_792402, EPI_ISL_792403, EPI_ISL_792404, EPI_ISL_792405, EPI_ISL_792406, EPI_ISL_792407, EPI_ISL_792408, EPI_ISL_792409, EPI_ISL_792410, EPI_ISL_792411, EPI_ISL_792412, EPI_ISL_792413, EPI_ISL_792414, EPI_ISL_792415, EPI_ISL_792416, EPI_ISL_792417, EPI_ISL_792418, EPI_ISL_792419, EPI_ISL_792420, EPI_ISL_792421, EPI_ISL_792422, EPI_ISL_792423			
see above	LABORATORIO DE CAMPAÑA COVID 19 INTA	Instituto de Biotecnología, IABIMO (CONICET), Instituto de Virología, IVIT(CONICET), Instituto de Patobiología, IPVET(CONICET), CICVyA, INTA on behalf of 'Proyecto Argentino Interinstitucional de genomica de SARS-CoV-2' (PAIS Consortium)	A; AF; AJ; AV; Asurmendi; B; Bengoa Luoni; Cacciabué; Chimeno; Craig; D; Del Médico; Delgado; Diagiacomo; Distéfano; Dus Santos; Díaz Carrasco; E; F; Farber; Fass; Ferández PC; Franco; Fusco; GA; Garbaccio; Gioffré; Gómez; Huertas; I; J; K; Klepp; König; L; LC; Lozano Calderón; Lucero; M; MD; MG; MI; MJ; MPD; MS; Muñoz Hidalgo; NB; Olivera; P; PA; Paniego; Pedroarias; Peralta; Perea; Pereda; Puebla; Rimondi; Rivarola; Rodríguez; Rosende; S; Schammas; Sioya; Soria; Trono; V; VC; Vera; Viegas, M.; Vissani; W; Zavallo; Zumárraga; Álvarez
EPI_ISL_430793, EPI_ISL_430794	Laboratorio Análisis Clínicos, Unidad de Servicios Diagnósticos, Swiss Medical Group	Área de Secuenciación del Laboratorio de Virología del Hospital de Niños Dr. Ricardo Gutierrez on behalf of 'Proyecto Argentino Interinstitucional de genomica de SARS-CoV-2' (PAIS Consortium)	AS; D; Goya; Guevara; LE; Lusso; MI; MS; Mistchenko; Nabaes Jodar; Natale; O; S; SM; Sanchez; Valinotto; Vicario; Viegas, M.
EPI_ISL_2449016	Laboratorio Central "Mg. Luis Alfredo Pianciola"	Área de Secuenciación del Laboratorio de Virología del Hospital de Niños Dr. Ricardo Gutierrez on behalf of 'Proyecto Argentino Interinstitucional de genomica de SARS-CoV-2' (PAIS Consortium)	A; Acuña; Alexay; C; D; Fernández; Goya; LE; Lusso; M; MC; MI; Mazzeo; Nabaes Jodar; Natale; Pianciola, L.; Pinto; S; Valinotto; Viegas, M.; Ziehm
EPI_ISL_1395932, EPI_ISL_1395933, EPI_ISL_1395934, EPI_ISL_1395935	Laboratorio Central De Redes y Programas	Grupo de Genómica y Bioinformática del Instituto de Investigación de la Cadena Láctea CONICET-INTA on behalf of 'Proyecto Argentino Interinstitucional de genomica de SARS-CoV-2' (PAIS Consortium)	AF; Amadio; Antonieta Cayré; Eberhardt; Gerardo Andino; Irazoqui; Laura Lescano; MF; Natalia Ruiz Diaz
EPI_ISL_792474, EPI_ISL_792475, EPI_ISL_792476, EPI_ISL_792477, EPI_ISL_792478, EPI_ISL_792479, EPI_ISL_792480, EPI_ISL_792481, EPI_ISL_792482, EPI_ISL_792483, EPI_ISL_792484, EPI_ISL_792485, EPI_ISL_792486, EPI_ISL_792487, EPI_ISL_792488, EPI_ISL_792489, EPI_ISL_792490, EPI_ISL_792491, EPI_ISL_792492, EPI_ISL_792493, EPI_ISL_792494, EPI_ISL_792495, EPI_ISL_792496, EPI_ISL_792497, EPI_ISL_792498, EPI_ISL_792499			
see above	Laboratorio Central Mg. Luis Alfredo Pianciola	Hospital Regional Ushuaia - Centro Austral De Investigaciones Científicas - Universidad Nacional De Tierra Del Fuego on behalf of 'Proyecto Argentino Interinstitucional de genomica de SARS-CoV-2' (PAIS Consortium)	C; CF; Ceballos; F; Gallego; Gramundi; ID; M; MC; Mazzeo; Nardi; Pianciola, L.; Pinto; SG; Ziehm
EPI_ISL_964887, EPI_ISL_964891, EPI_ISL_964893, EPI_ISL_964894, EPI_ISL_964895, EPI_ISL_964896, EPI_ISL_964898, EPI_ISL_964899, EPI_ISL_964900, EPI_ISL_964902, EPI_ISL_964903, EPI_ISL_964904, EPI_ISL_964905, EPI_ISL_964906, EPI_ISL_964907, EPI_ISL_964908, EPI_ISL_964909, EPI_ISL_964910, EPI_ISL_964911, EPI_ISL_964912, EPI_ISL_964914, EPI_ISL_981053			
see above	Laboratorio Central Mg. Luis Alfredo Pianciola on behalf of 'Proyecto Argentino Interinstitucional de genomica de SARS-CoV-2' (PAIS Consortium)	Laboratorio Central Mg. Luis Alfredo Pianciola on behalf of 'Proyecto Argentino Interinstitucional de genomica de SARS-CoV-2' (PAIS Consortium)	C Pintos; C Ziehm; J Ousset; L Pianciola.; M Fernandez; M Mazzeo; M Nabaes

EPI_ISL_981030, EPI_ISL_981031, EPI_ISL_981032, EPI_ISL_981033, EPI_ISL_981034, EPI_ISL_981035, EPI_ISL_981036, EPI_ISL_981037, EPI_ISL_981038, EPI_ISL_981039, EPI_ISL_981040, EPI_ISL_981041, EPI_ISL_981042, EPI_ISL_981043, EPI_ISL_981044, EPI_ISL_981045, EPI_ISL_981046, EPI_ISL_981047, EPI_ISL_981048, EPI_ISL_981049, EPI_ISL_981050, EPI_ISL_981051, EPI_ISL_981052, EPI_ISL_981054, EPI_ISL_981055, EPI_ISL_981056			
see above	Laboratorio Central Sección Virología. Hospital Regional Artemides Zatti (Viedma, Rio Negro); Laboratorio MICROBIOM (General Roca, Río Negro); Hospital Zonal Dr. Ramón Carrillo (San Carlos de Bariloche, Río Negro)	Laboratorio Central Mg. Luis Alfredo Pianciola on behalf of 'Proyecto Argentino Interinstitucional de genomica de SARS-CoV-2' (PAIS Consortium)	Antonela De Fino; C Pintos; C Ziehm; Darío Fabián di Prátula; J Ousset; L Pianciola.; Liliana Fonseca; M Fernandez; M Mazzeo; M Nabaes; Marcela Nóbile; María Laura Álvarez; Patricia Valeria Blanco; Silvana Cecchi; Yesica Espasandin
EPI_ISL_2363550	Laboratorio Central de Redes y Programas de Corrientes	Grupo de Genómica y Bioinformática del Instituto de Investigación de la Cadena Láctea CONICET-INTA on behalf of 'Proyecto Argentino Interinstitucional de genomica de SARS-CoV-2' (PAIS Consortium)	A; AF; Amadio; Andino; Cayré; Eberhardt; G; Irazoqui; JM; L; Lescano; MF; N; Ruiz Diaz
EPI_ISL_1395937, EPI_ISL_1395938, EPI_ISL_1395940, EPI_ISL_1395941, EPI_ISL_1395942, EPI_ISL_1395943, EPI_ISL_1395944, EPI_ISL_1395945, EPI_ISL_1395946, EPI_ISL_1395947, EPI_ISL_1395948, EPI_ISL_1395949, EPI_ISL_1395950, EPI_ISL_1395951, EPI_ISL_1395952, EPI_ISL_1395953, EPI_ISL_1395954, EPI_ISL_1395955, EPI_ISL_1395956, EPI_ISL_1395957, EPI_ISL_1395958			
see above	Laboratorio Central de Salud Pública	Grupo de Genómica y Bioinformática del Instituto de Investigación de la Cadena Láctea CONICET-INTA on behalf of 'Proyecto Argentino Interinstitucional de genomica de SARS-CoV-2' (PAIS Consortium)	AF; Amadio; Antonieta Cayré; Eberhardt; Erica Struss; Esteban Paredes; Irazoqui; Laura Lescano; MF; Natalia Andrea Ayala y María Verónica Gómez
EPI_ISL_1395959, EPI_ISL_1395960, EPI_ISL_1395961, EPI_ISL_1395962, EPI_ISL_1395963, EPI_ISL_1395964, EPI_ISL_1395965, EPI_ISL_1395966, EPI_ISL_2940196, EPI_ISL_2940198, EPI_ISL_2940199, EPI_ISL_2940201, EPI_ISL_2940202, EPI_ISL_2940204, EPI_ISL_2940205			
see above	Laboratorio Central de Salud Pública de la Provincia de Jujuy	Instituto de Patologia Vegetal (CIAP-INTA) on behalf of 'Proyecto Argentino Interinstitucional de genomica de SARS-CoV-2' (PAIS Consortium)	A. Miguel Alejandro Charre; Amadio; Ariel David Fridman; Claudia Mamani; Debat, HJ.; FD; Fabiana Vaca; Fabiana Vaca.; Fernández; Irazoqui, M.; Marquez, N.; Miguel Alejandro Charre
EPI_ISL_792512, EPI_ISL_792513, EPI_ISL_792514, EPI_ISL_792515, EPI_ISL_792516, EPI_ISL_792517, EPI_ISL_792518, EPI_ISL_792519, EPI_ISL_792520, EPI_ISL_792521, EPI_ISL_1395840, EPI_ISL_1395841, EPI_ISL_1395842, EPI_ISL_1395843, EPI_ISL_1395844, EPI_ISL_1395845, EPI_ISL_1395846, EPI_ISL_1395847, EPI_ISL_1395848, EPI_ISL_1395849, EPI_ISL_1395850, EPI_ISL_1395851, EPI_ISL_1395852, EPI_ISL_1395853, EPI_ISL_1395854, EPI_ISL_1395855, EPI_ISL_1395856, EPI_ISL_1395857, EPI_ISL_1395858, EPI_ISL_1395859, EPI_ISL_1395860, EPI_ISL_1395861, EPI_ISL_1395862, EPI_ISL_1395863, EPI_ISL_1395864, EPI_ISL_1395865, EPI_ISL_1395866, EPI_ISL_1395867, EPI_ISL_1395868, EPI_ISL_1395869, EPI_ISL_1395870, EPI_ISL_1395871, EPI_ISL_1395872, EPI_ISL_1395873, EPI_ISL_1395874, EPI_ISL_1395875, EPI_ISL_1395876, EPI_ISL_1395877, EPI_ISL_1395878, EPI_ISL_1395879, EPI_ISL_1395880, EPI_ISL_1395881, EPI_ISL_1395882, EPI_ISL_1395883, EPI_ISL_1395884, EPI_ISL_1395885, EPI_ISL_1395886, EPI_ISL_1395887, EPI_ISL_1395888, EPI_ISL_1395889, EPI_ISL_1395890, EPI_ISL_1395891, EPI_ISL_1395892, EPI_ISL_1395893, EPI_ISL_1395894, EPI_ISL_1395895, EPI_ISL_1395896, EPI_ISL_1395897, EPI_ISL_1395898, EPI_ISL_1395899, EPI_ISL_1395900, EPI_ISL_1395901, EPI_ISL_1395902, EPI_ISL_1395903, EPI_ISL_1395904, EPI_ISL_1395905, EPI_ISL_1395906, EPI_ISL_1395907, EPI_ISL_1395908, EPI_ISL_1395909, EPI_ISL_1395910, EPI_ISL_1395911, EPI_ISL_1395912, EPI_ISL_1395913, EPI_ISL_1395914, EPI_ISL_1395915, EPI_ISL_1395916, EPI_ISL_1395917, EPI_ISL_1395918, EPI_ISL_1395919, EPI_ISL_1395920, EPI_ISL_1395921, EPI_ISL_1395922, EPI_ISL_1395923, EPI_ISL_1395924, EPI_ISL_1395925, EPI_ISL_1395926, EPI_ISL_1395927, EPI_ISL_1395928, EPI_ISL_1395929, EPI_ISL_1395930, EPI_ISL_1395931			
see above	Laboratorio Central de la Ciudad de Santa Fe	Grupo de Genómica y Bioinformática del Instituto de Investigación de la Cadena Láctea CONICET-INTA on behalf of 'Proyecto Argentino Interinstitucional de genomica de SARS-CoV-2' (PAIS Consortium)	AF; Amadio; C; Eberhardt; G; Irazoqui; JM; MF; Mugna; Ojeda; Pastor; Rompato; V
EPI_ISL_1395967, EPI_ISL_1395968, EPI_ISL_1395969, EPI_ISL_1395970, EPI_ISL_1395971, EPI_ISL_1395972, EPI_ISL_1395973, EPI_ISL_1395974, EPI_ISL_1395975, EPI_ISL_1395976, EPI_ISL_1395977, EPI_ISL_1395978, EPI_ISL_1395979, EPI_ISL_1395980, EPI_ISL_1395981, EPI_ISL_1395982, EPI_ISL_1395983, EPI_ISL_1395984, EPI_ISL_1395985, EPI_ISL_1395986, EPI_ISL_1395987, EPI_ISL_1395988, EPI_ISL_1395989, EPI_ISL_1395993, EPI_ISL_1395994, EPI_ISL_1395995, EPI_ISL_1395996, EPI_ISL_1395997, EPI_ISL_1395998, EPI_ISL_1395999, EPI_ISL_1396000, EPI_ISL_1396001, EPI_ISL_1396002, EPI_ISL_1396003, EPI_ISL_1396004, EPI_ISL_1396005, EPI_ISL_1396006, EPI_ISL_1396007, EPI_ISL_1396008, EPI_ISL_1396009, EPI_ISL_1396010, EPI_ISL_1396011, EPI_ISL_1396012, EPI_ISL_1396013, EPI_ISL_1396014, EPI_ISL_1396015, EPI_ISL_1396016, EPI_ISL_1396017, EPI_ISL_1396018, EPI_ISL_1396019, EPI_ISL_1396020, EPI_ISL_1396021, EPI_ISL_1396022, EPI_ISL_1396023, EPI_ISL_1396024, EPI_ISL_1396025, EPI_ISL_1396026, EPI_ISL_1396027, EPI_ISL_1396028, EPI_ISL_1396029, EPI_ISL_1396031, EPI_ISL_1396032, EPI_ISL_1396033, EPI_ISL_1396034, EPI_ISL_1396035, EPI_ISL_1396036, EPI_ISL_1396037, EPI_ISL_1396038, EPI_ISL_1396039, EPI_ISL_1396040, EPI_ISL_1396041, EPI_ISL_1396042, EPI_ISL_1396043, EPI_ISL_1396044, EPI_ISL_1396045, EPI_ISL_1396046, EPI_ISL_1396048, EPI_ISL_1396049, EPI_ISL_1396050, EPI_ISL_1396051, EPI_ISL_1396052, EPI_ISL_1396053, EPI_ISL_1396054, EPI_ISL_1396055, EPI_ISL_1396056, EPI_ISL_1396057, EPI_ISL_1396058, EPI_ISL_1396059, EPI_ISL_1396060			
see above	Laboratorio Central, Ministerio de Salud Cordoba	Instituto de Patologia Vegetal (CIAP-INTA) on behalf of 'Proyecto Argentino Interinstitucional de genomica de SARS-CoV-2' (PAIS Consortium)	Barbas, G.; Castro, G.; Debat, HJ.; FD; Fernández; M.B.; Marquez, N.; Pisano; Re, V.
EPI_ISL_792526, EPI_ISL_792527, EPI_ISL_792528, EPI_ISL_792529, EPI_ISL_792530, EPI_ISL_792531, EPI_ISL_792532, EPI_ISL_792533, EPI_ISL_792534, EPI_ISL_792535, EPI_ISL_792536, EPI_ISL_792537, EPI_ISL_792538, EPI_ISL_792539, EPI_ISL_792540, EPI_ISL_792541, EPI_ISL_792542, EPI_ISL_792543, EPI_ISL_792544, EPI_ISL_792545, EPI_ISL_792546			
see above	Laboratorio Central, Ministerio de Salud Córdoba	Instituto de Patologia Vegetal (CIAP-INTA) on behalf of 'Proyecto Argentino Interinstitucional de genomica de SARS-CoV-2' (PAIS Consortium)	Barbas, G.; Castro, G.; Debat, HJ.; FD; Fernández; MB; Pisano; Re; V
EPI_ISL_1396383, EPI_ISL_1396384, EPI_ISL_1396385, EPI_ISL_1396386, EPI_ISL_1396387, EPI_ISL_1396388, EPI_ISL_1396389, EPI_ISL_1396390			
see above	Laboratorio Salud Publica Mendoza	Nodo de Secuenciación Tierra del Fuego - Hospital Regional Ushuaia - Centro Austral De Investigaciones Científicas - Universidad Nacional De Tierra Del Fuego on behalf of 'Proyecto Argentino Interinstitucional de genomica de SARS-CoV-2' (PAIS Consortium)	Aguirre Carolina; Alberto Carena; Alejandro Ezequiel Rojas; Andrea Falaschi; Belén Ortiz; Bosio Lia; Carlos Espul; Cristian Garay; Cristina Fernanda Nardi; Fernando Gallego; Fernando Giuliani; Ivan Dario Gramundi; Luciana Martinez; Maria Belen Peralta Roca; Patricia Robledo; Santiago Guillermo Ceballos; Silvia Zerrer; Silvina Denita
EPI_ISL_842652	Laboratorio de Biología Molecular Hospital Pedro de Elizalde	Grupo de Genómica y Bioinformática del Instituto de Investigación de la Cadena Láctea CONICET-INTA on behalf of 'Proyecto Argentino Interinstitucional de genomica de SARS-CoV-2' (PAIS Consortium)	A; AF; Alegre; Alexay; Amadio; Aulicino; B; Bressan; C; Chamorro; Claps; D; Diaz; E; Eberhardt; F; FJ; G; Gondolessi; Goya; Gómez; Indart; Irazoqui; J; König; L; Lorenzo; Lusso; M; ME; MF; ML; MS; Marchetti; Martín; Montoto Piazza; Morandi; N; Nabaes Jodar; Natale; Osaba; P; Paez; Rocovich; Rosales; S; Sanchez; Sueiro; Torres; Valinotto; Viegas, M.; Wenk; Zamora
EPI_ISL_1394885	Laboratorio de Biología Molecular Hospital Pedro de Elizalde	Área de Secuenciación del Laboratorio de Virología del Hospital de Niños Dr. Ricardo Gutierrez on behalf of 'Proyecto Argentino Interinstitucional de genomica de SARS-CoV-2' (PAIS Consortium)	Acuña; Aldana Claps; Alexay; Belen Carolina Diaz; D; Daiana Rosales; Emilce osaba; Eugenia Paez; Federico José Torres; Florencia Alegre; Franco Morandi; Goya; Gretel Wenk; Jaqueline Rocovich; Javier Indart; Julieta Gondolessi; Julián Chamorro; LE; Laura Bressan; Luciana Montoto Piazza; Lusso; M; MI; Maria Laura Sueiro; Marisa Lorena Gómez; María Eugenia Martín; María Florencia Sanchez; Nabaes Jodar; Natale; Natalia Zamora; Paulina Marchetti; S; Valinotto; Viegas, M.
EPI_ISL_792354, EPI_ISL_792355, EPI_ISL_792356, EPI_ISL_792357	Laboratorio de Biología Molecular. Hospital Dr. Héctor Cura	Área de Secuenciación del Laboratorio de Virología del Hospital de Niños Dr. Ricardo Gutierrez on behalf of 'Proyecto Argentino Interinstitucional de genomica de SARS-CoV-2' (PAIS Consortium)	Ghiano; Goya; J; LE; Lusso; MB; MI; MS; N; Nabaes Jodar; Natale; R; S; Spina; Turrina; Valinotto; Viegas, M.; Zaffanella
EPI_ISL_1394925, EPI_ISL_1394926, EPI_ISL_1394927, EPI_ISL_1394928, EPI_ISL_1394930, EPI_ISL_1394931, EPI_ISL_1394933, EPI_ISL_1394934, EPI_ISL_1394935, EPI_ISL_1395055			
see above	Laboratorio de Diagnóstico de la UNAHUR	Área de Secuenciación del Laboratorio de Virología del Hospital de Niños Dr. Ricardo Gutierrez on behalf of 'Proyecto Argentino Interinstitucional de genomica de SARS-CoV-2' (PAIS Consortium)	Acuña; Adriana Fernandez Souto; Aldana Trotta; Alexay; Angel German Arias; Angélica María Ramirez Londoño; Blanc Daiana Sofia; Boero Carolina Jazmín; Brenda Gimeno; Camila Frydman; Cristina Belén Serrano; D; Daniela Vega; David Ybarra; Didier Garnham Mercedes; Germán Albornoz; Goya; Juan Manuel Velazquez; LE; Luis Castillo; Lusso; M; MI; Marcela Pilloff; Maria Natalia Calienni; Maria del C Lorenzo; Marina Mozgovoj; María José Dus Santos; Nabaes Jodar; Natale; Pablo Raies; S; Sabrina Amalfi; Valeria Marsal; Valinotto; Vanina Saraullo; Viegas, M.
EPI_ISL_792500, EPI_ISL_792501, EPI_ISL_792502, EPI_ISL_792503, EPI_ISL_792504, EPI_ISL_792505, EPI_ISL_792506, EPI_ISL_792507, EPI_ISL_792508, EPI_ISL_792509, EPI_ISL_792510, EPI_ISL_792511			
see above	Laboratorio de Inmunología del Hospital Perrando e Instituto de Medicina Regional de la UNNE	Grupo de Genómica y Bioinformática del Instituto de Investigación de la Cadena Láctea CONICET-INTA on behalf of 'Proyecto Argentino Interinstitucional de genomica de SARS-CoV-2' (PAIS Consortium)	A; AF; Amadio; Ayala; Cayré; Deluca; Eberhardt; Foussal; G; Giusiano; Gómez; H; Irazoqui; JM; L; Lescano; Lucero; M; MD; MF; MV; Marin; NA
EPI_ISL_792424, EPI_ISL_792425, EPI_ISL_792426, EPI_ISL_792427, EPI_ISL_792428, EPI_ISL_792429, EPI_ISL_792430, EPI_ISL_792431, EPI_ISL_792432, EPI_ISL_792433, EPI_ISL_792434, EPI_ISL_792435, EPI_ISL_792436, EPI_ISL_792437, EPI_ISL_792438, EPI_ISL_792439, EPI_ISL_792440, EPI_ISL_792441, EPI_ISL_792442			
see above	Laboratorio de Inmunología del Hospital Perrando e Instituto de Medicina Regional de la UNNE	Instituto de Biotecnología, IABIMO (CONICET), Instituto de Virología, IVIT(CONICET), Instituto de Patobiología, IPVET(CONICET), CICVyA, INTA on behalf of 'Proyecto Argentino Interinstitucional de genomica de SARS-CoV-2' (PAIS Consortium)	A; AF; AJ; AV; Asurmendi; Ayala; Bengoa Luoni; Cacciabué; Cayré; D; Deluca; Distéfano; Farber; Fass; Foussal; G; GA; Giusiano; Gómez; H; König; L; LC; Lescano; Lozano Calderón; Lucero; M; MD; MG; MPD; MV; Marin; Muñoz Hidalgo; NA; NB; PA; Paniego; Pedroarias; Peralta; Puebla; Rivarola; S; VC; Vera; Viegas, M.; Zavallo
EPI_ISL_1396061, EPI_ISL_1396062, EPI_ISL_1396063, EPI_ISL_1396064, EPI_ISL_1396065, EPI_ISL_1396066, EPI_ISL_1396067, EPI_ISL_1396068, EPI_ISL_1396069, EPI_ISL_1396070, EPI_ISL_1396071, EPI_ISL_1396072, EPI_ISL_1396073, EPI_ISL_1396074, EPI_ISL_1396075, EPI_ISL_1396076, EPI_ISL_1396077, EPI_ISL_1396078, EPI_ISL_1396079, EPI_ISL_1396080, EPI_ISL_1396081, EPI_ISL_1396082, EPI_ISL_1396083, EPI_ISL_1396084, EPI_ISL_1396085, EPI_ISL_1396086, EPI_ISL_1396087, EPI_ISL_1396088, EPI_ISL_1396089, EPI_ISL_1396090, EPI_ISL_1396091			
see above	Laboratorio de Salud Pública	Instituto de Patologia Vegetal (CIAP-INTA) on behalf of 'Proyecto Argentino Interinstitucional de genomica de SARS-CoV-2' (PAIS Consortium)	A. Mariana B. Salmerón; Amadio; Ana Maria Zamora; Dardo E. Costas; Debat, HJ.; FD; Fernández; Graciela Alabarse.; Gustavo Ruìz de Huidobro; Irazoqui, M.; Marquez, N.
EPI_ISL_1394932, EPI_ISL_1394952, EPI_ISL_1394953, EPI_ISL_1395070	Laboratorio de Virologia - HIEAyC "San Juan de Dios"	Área de Secuenciación del Laboratorio de Virología del Hospital de Niños Dr. Ricardo Gutierrez on behalf of 'Proyecto Argentino Interinstitucional de genomica de SARS-CoV-2' (PAIS Consortium)	Acuña; Alexay; Andrea Gatelli; D; Francisco Echeverria; Goya; Karina Gil; LE; Lusso; M; MI; Maria Colmeiro; Martina Ferioli; Nabaes Jodar; Natale; Ramiro Agüero; Regina Ercole; S; Silvia Galvez; Valinotto; Viegas, M.
EPI_ISL_792358, EPI_ISL_792359, EPI_ISL_792360, EPI_ISL_792361, EPI_ISL_792362, EPI_ISL_792363, EPI_ISL_792364, EPI_ISL_792365, EPI_ISL_792366, EPI_ISL_792367, EPI_ISL_792368, EPI_ISL_792369, EPI_ISL_792370			
see above	Laboratorio de Virologia - HIEAyC San Juan de Dios	Área de Secuenciación del Laboratorio de Virología del Hospital de Niños Dr. Ricardo Gutierrez on behalf of 'Proyecto Argentino Interinstitucional de genomica de SARS-CoV-2' (PAIS Consortium)	A; Colmeiro; Ercole; Ferioli; Gatelli; Goya; LE; Lusso; M; MI; MS; Nabaes Jodar; Natale; R; S; Valinotto; Viegas, M.
EPI_ISL_792397, EPI_ISL_792398, EPI_ISL_792399, EPI_ISL_792400, EPI_ISL_792401, EPI_ISL_1396246, EPI_ISL_1396247, EPI_ISL_1396248, EPI_ISL_1396249, EPI_ISL_1396250, EPI_ISL_1396251, EPI_ISL_1396252, EPI_ISL_1396253, EPI_ISL_1396254, EPI_ISL_1396255, EPI_ISL_1396256, EPI_ISL_1396257, EPI_ISL_1396258, EPI_ISL_1396259, EPI_ISL_1396260, EPI_ISL_1396261, EPI_ISL_1396262, EPI_ISL_1396263, EPI_ISL_1396264, EPI_ISL_1396265, EPI_ISL_1396266, EPI_ISL_1396267, EPI_ISL_1396268, EPI_ISL_1396269, EPI_ISL_1396270, EPI_ISL_1396271, EPI_ISL_1396272, EPI_ISL_1396273, EPI_ISL_1396274, EPI_ISL_1396275, EPI_ISL_1396276, EPI_ISL_1396277, EPI_ISL_1396278, EPI_ISL_1396279, EPI_ISL_1396280, EPI_ISL_1396281, EPI_ISL_1396282, EPI_ISL_1396283, EPI_ISL_1396284, EPI_ISL_1396285, EPI_ISL_1396286, EPI_ISL_1396287, EPI_ISL_1396288, EPI_ISL_1396289, EPI_ISL_1396290, EPI_ISL_1396291, EPI_ISL_1396292, EPI_ISL_1396293, EPI_ISL_1396294, EPI_ISL_1396295, EPI_ISL_1396296, EPI_ISL_1396297, EPI_ISL_1396298, EPI_ISL_1396299, EPI_ISL_1396300, EPI_ISL_1396301, EPI_ISL_1396302, EPI_ISL_1396303, EPI_ISL_1396304, EPI_ISL_1396305, EPI_ISL_1396306, EPI_ISL_1396307, EPI_ISL_1396308, EPI_ISL_1396309, EPI_ISL_1396310, EPI_ISL_1396311, EPI_ISL_1396312, EPI_ISL_1396313, EPI_ISL_1396314, EPI_ISL_1396315, EPI_ISL_1396316, EPI_ISL_1396317, EPI_ISL_1396318, EPI_ISL_1396319, EPI_ISL_1396320, EPI_ISL_1396321, EPI_ISL_1396322, EPI_ISL_1396323, EPI_ISL_1396324, EPI_ISL_1396325, EPI_ISL_1396326, EPI_ISL_1396327, EPI_ISL_1396328, EPI_ISL_1396329, EPI_ISL_1396330, EPI_ISL_1396331			
see above	Laboratorio de Virología del Hospital de Niños Dr. Ricardo Gutierrez	Biocódices SA. on behalf of 'Proyecto Argentino Interinstitucional de genomica de SARS-CoV-2' (PAIS Consortium)	A; Acevedo; Alexay; Alvarez Lopez; Barreda Frank; Berros; Berros JM; C; Dopazo H; Dopazo, H.; E; G; Grandis; Gravis; J; JM; Jacques; Labarta; M; ME; Medina; Mistchenko; N; O; S; Streitenberger; Thomas; Villegas; Zubrzycki J
EPI_ISL_792522, EPI_ISL_792523, EPI_ISL_792524	Laboratorio de Virología del Hospital de Niños Dr. Ricardo Gutierrez	Grupo de Genómica y Bioinformática del Instituto de Investigación de la Cadena Láctea CONICET-INTA on behalf of 'Proyecto Argentino Interinstitucional de genomica de SARS-CoV-2' (PAIS Consortium)	AF; AS; Acevedo; Alexay; Alvarez Lopez; Amadio; Aulicino; C; Eberhardt; G; Goya; Irazoqui; Jacques; König; M; ME; MF; MS; Mistchenko; Nabaes Jodar; O; P; S; Torres; Viegas, M.
EPI_ISL_430795, EPI_ISL_430799, EPI_ISL_430800, EPI_ISL_430801, EPI_ISL_430803, EPI_ISL_430804, EPI_ISL_430807, EPI_ISL_430809, EPI_ISL_430810, EPI_ISL_430811, EPI_ISL_430812, EPI_ISL_430813, EPI_ISL_430814, EPI_ISL_430815, EPI_ISL_430816, EPI_ISL_430817, EPI_ISL_430818, EPI_ISL_792117, EPI_ISL_792118, EPI_ISL_792119, EPI_ISL_792120, EPI_ISL_792121, EPI_ISL_792122, EPI_ISL_792123, EPI_ISL_792124, EPI_ISL_792125, EPI_ISL_792126, EPI_ISL_792127, EPI_ISL_792128, EPI_ISL_792129, EPI_ISL_792130, EPI_ISL_792131, EPI_ISL_792132, EPI_ISL_792133, EPI_ISL_792134, EPI_ISL_792135, EPI_ISL_792136, EPI_ISL_792137, EPI_ISL_792138, EPI_ISL_792139, EPI_ISL_792140, EPI_ISL_792141, EPI_ISL_792142, EPI_ISL_792143, EPI_ISL_792144, EPI_ISL_792145, EPI_ISL_792146, EPI_ISL_792147, EPI_ISL_792148, EPI_ISL_792149, EPI_ISL_792150, EPI_ISL_792151, EPI_ISL_792152, EPI_ISL_792153, EPI_ISL_792154, EPI_ISL_792155, EPI_ISL_792156, EPI_ISL_792157, EPI_ISL_792158, EPI_ISL_792159, EPI_ISL_792160, EPI_ISL_792161, EPI_ISL_792162, EPI_ISL_792163, EPI_ISL_792164, EPI_ISL_792165, EPI_ISL_792166, EPI_ISL_792167, EPI_ISL_792168, EPI_ISL_792169, EPI_ISL_792170, EPI_ISL_792171, EPI_ISL_792172, EPI_ISL_792173, EPI_ISL_792174, EPI_ISL_792175, EPI_ISL_792176, EPI_ISL_792177, EPI_ISL_792178, EPI_ISL_792179, EPI_ISL_792180, EPI_ISL_792181, EPI_ISL_792182, EPI_ISL_792183, EPI_ISL_792184, EPI_ISL_792185, EPI_ISL_792186, EPI_ISL_792187, EPI_ISL_792188, EPI_ISL_792189, EPI_ISL_792190, EPI_ISL_792191, EPI_ISL_792192, EPI_ISL_792193, EPI_ISL_792194, EPI_ISL_792195, EPI_ISL_792196, EPI_ISL_792197, EPI_ISL_792198, EPI_ISL_792199, EPI_ISL_792200, EPI_ISL_792201, EPI_ISL_792202, EPI_ISL_792203, EPI_ISL_792204, EPI_ISL_792205, EPI_ISL_792206, EPI_ISL_792207, EPI_ISL_792208, EPI_ISL_792209, EPI_ISL_792210, EPI_ISL_792211, EPI_ISL_792212, EPI_ISL_792213, EPI_ISL_792214, EPI_ISL_792215, EPI_ISL_792216, EPI_ISL_792217, EPI_ISL_792218, EPI_ISL_792219, EPI_ISL_792220, EPI_ISL_792221, EPI_ISL_792222, EPI_ISL_792223, EPI_ISL_792224, EPI_ISL_792225, EPI_ISL_792226, EPI_ISL_792227, EPI_ISL_792228, EPI_ISL_792229, EPI_ISL_792230, EPI_ISL_792231, EPI_ISL_792232, EPI_ISL_792233, EPI_ISL_792234, EPI_ISL_792235, EPI_ISL_792236, EPI_ISL_792237, EPI_ISL_792238, EPI_ISL_792239, EPI_ISL_792240, EPI_ISL_792241, EPI_ISL_792242, EPI_ISL_792243, EPI_ISL_792244, EPI_ISL_792245, EPI_ISL_792246, EPI_ISL_792247, EPI_ISL_792248, EPI_ISL_792249, EPI_ISL_792250, EPI_ISL_792251, EPI_ISL_792252, EPI_ISL_792253, EPI_ISL_792254, EPI_ISL_792255, EPI_ISL_792256, EPI_ISL_792257, EPI_ISL_792258, EPI_ISL_792259, EPI_ISL_792260, EPI_ISL_792261, EPI_ISL_792262, EPI_ISL_792263, EPI_ISL_792264, EPI_ISL_792265, EPI_ISL_792266, EPI_ISL_792267, EPI_ISL_792268, EPI_ISL_792269, EPI_ISL_792270, EPI_ISL_792271, EPI_ISL_792272, EPI_ISL_792273, EPI_ISL_792274, EPI_ISL_792275, EPI_ISL_792276, EPI_ISL_792277, EPI_ISL_792278, EPI_ISL_792279, EPI_ISL_792280, EPI_ISL_792281, EPI_ISL_792282, EPI_ISL_792283, EPI_ISL_792284, EPI_ISL_792285, EPI_ISL_792286, EPI_ISL_792287, EPI_ISL_792288, EPI_ISL_792289, EPI_ISL_792290, EPI_ISL_793013, EPI_ISL_1394870, EPI_ISL_1394871, EPI_ISL_1394872, EPI_ISL_1394873, EPI_ISL_1394874, EPI_ISL_1394875, EPI_ISL_1394876, EPI_ISL_1394877, EPI_ISL_1394878, EPI_ISL_1394879, EPI_ISL_1394880, EPI_ISL_1394881, EPI_ISL_1394882, EPI_ISL_1394883, EPI_ISL_1394884, EPI_ISL_1394886, EPI_ISL_1394887, EPI_ISL_1394888, EPI_ISL_1394889, EPI_ISL_1394890, EPI_ISL_1394891, EPI_ISL_1394892, EPI_ISL_1394893, EPI_ISL_1394894, EPI_ISL_1394895, EPI_ISL_1394896, EPI_ISL_1394897, EPI_ISL_1394898, EPI_ISL_1394899, EPI_ISL_1394900, EPI_ISL_1394901, EPI_ISL_1394902, EPI_ISL_1394903, EPI_ISL_1394904, EPI_ISL_1394905, EPI_ISL_1394906, EPI_ISL_1394907, EPI_ISL_1394941, EPI_ISL_1394942, EPI_ISL_1394943, EPI_ISL_1394944, EPI_ISL_1394947, EPI_ISL_1394950, EPI_ISL_1394951, EPI_ISL_1394954, EPI_ISL_1394955, EPI_ISL_1394956, EPI_ISL_1394957, EPI_ISL_1394958, EPI_ISL_1394959, EPI_ISL_1394960, EPI_ISL_1394961, EPI_ISL_1394962, EPI_ISL_1394963, EPI_ISL_1394964, EPI_ISL_1394965, EPI_ISL_1394966, EPI_ISL_1394967, EPI_ISL_1394968, EPI_ISL_1394969, EPI_ISL_1394970, EPI_ISL_1394971, EPI_ISL_1394973, EPI_ISL_1394974, EPI_ISL_1394975, EPI_ISL_1394976,			

EPI_ISL_1394977, EPI_ISL_1394978, EPI_ISL_1394979, EPI_ISL_1394980, EPI_ISL_1394981, EPI_ISL_1394982, EPI_ISL_1394983, EPI_ISL_1394984, EPI_ISL_1394986, EPI_ISL_1394987, EPI_ISL_1394988, EPI_ISL_1394989, EPI_ISL_1394990, EPI_ISL_1394991, EPI_ISL_1394992, EPI_ISL_1394993, EPI_ISL_1394994, EPI_ISL_1394995, EPI_ISL_1394996, EPI_ISL_1394997, EPI_ISL_1395001, EPI_ISL_1395002, EPI_ISL_1395003, EPI_ISL_1395004, EPI_ISL_1395005, EPI_ISL_1395006, EPI_ISL_1395007, EPI_ISL_1395008, EPI_ISL_1395009, EPI_ISL_1395010, EPI_ISL_1395011, EPI_ISL_1395012, EPI_ISL_1395013, EPI_ISL_1395014, EPI_ISL_1395015, EPI_ISL_1395016, EPI_ISL_1395021, EPI_ISL_1395022, EPI_ISL_1395023, EPI_ISL_1395024, EPI_ISL_1395025, EPI_ISL_1395026, EPI_ISL_1395027, EPI_ISL_1395028, EPI_ISL_1395029, EPI_ISL_1395030, EPI_ISL_1395031, EPI_ISL_1395032, EPI_ISL_1395033, EPI_ISL_1395034, EPI_ISL_1395035, EPI_ISL_1395042, EPI_ISL_1395043, EPI_ISL_1395044, EPI_ISL_1395045, EPI_ISL_1395046, EPI_ISL_1395047, EPI_ISL_1395048, EPI_ISL_1395049, EPI_ISL_1395050, EPI_ISL_1395051, EPI_ISL_1395056, EPI_ISL_1395057, EPI_ISL_1395058, EPI_ISL_1395059, EPI_ISL_1395060, EPI_ISL_1395061, EPI_ISL_1395062, EPI_ISL_1395063, EPI_ISL_1395064, EPI_ISL_1395065, EPI_ISL_1395066, EPI_ISL_1395071, EPI_ISL_1395777, EPI_ISL_1396149, EPI_ISL_1396150, EPI_ISL_1396151, EPI_ISL_1396152, EPI_ISL_1396153, EPI_ISL_1396154, EPI_ISL_1396155, EPI_ISL_1396156, EPI_ISL_1396157, EPI_ISL_1396158, EPI_ISL_1396159, EPI_ISL_1396160, EPI_ISL_1396161, EPI_ISL_1396162, EPI_ISL_1396163, EPI_ISL_1396164, EPI_ISL_1396165, EPI_ISL_1396166, EPI_ISL_1396167, EPI_ISL_1396168, EPI_ISL_1396169, EPI_ISL_1396170, EPI_ISL_1396171, EPI_ISL_1396172, EPI_ISL_1396173, EPI_ISL_1396174, EPI_ISL_1396175, EPI_ISL_1396176, EPI_ISL_1396177, EPI_ISL_1396178, EPI_ISL_1396179, EPI_ISL_1396180, EPI_ISL_1396181, EPI_ISL_1396182, EPI_ISL_1396183, EPI_ISL_1396184, EPI_ISL_1396185, EPI_ISL_1396186, EPI_ISL_1396187, EPI_ISL_1396188, EPI_ISL_1396189, EPI_ISL_1396190, EPI_ISL_1396191, EPI_ISL_1396192, EPI_ISL_1396193, EPI_ISL_1396194, EPI_ISL_1396195, EPI_ISL_1396196, EPI_ISL_1396197, EPI_ISL_1396198, EPI_ISL_1396199, EPI_ISL_1396200, EPI_ISL_1396201, EPI_ISL_1396202, EPI_ISL_1396203, EPI_ISL_1396204, EPI_ISL_1396205, EPI_ISL_1396206, EPI_ISL_1396207, EPI_ISL_1396208, EPI_ISL_1396209, EPI_ISL_1396210, EPI_ISL_1396211, EPI_ISL_1396212, EPI_ISL_1396213, EPI_ISL_1396214, EPI_ISL_1396215, EPI_ISL_1396216, EPI_ISL_1396217, EPI_ISL_1396218, EPI_ISL_1396219, EPI_ISL_1396220, EPI_ISL_1396221, EPI_ISL_1396222, EPI_ISL_1396223, EPI_ISL_1396226, EPI_ISL_1396227, EPI_ISL_1396228, EPI_ISL_1396229, EPI_ISL_1396230, EPI_ISL_1396231, EPI_ISL_1396232, EPI_ISL_1396233, EPI_ISL_1396234, EPI_ISL_1396235, EPI_ISL_1396236, EPI_ISL_1396237, EPI_ISL_1396238, EPI_ISL_1396239, EPI_ISL_1396240, EPI_ISL_1396241, EPI_ISL_1396242, EPI_ISL_1396243, EPI_ISL_1396244, EPI_ISL_1396245			
see above	Laboratorio de Virología del Hospital de Niños Dr. Ricardo Gutierrez	Área de Secuenciación del Laboratorio de Virología del Hospital de Niños Dr. Ricardo Gutierrez on behalf of 'Proyecto Argentino Interinstitucional de genomica de SARS-CoV-2' (PAIS Consortium)	A; AS; Acevedo; Acuña; Alexay; Alvarez Lopez; Barreda Frank; C; D; E; Echavarria; G; Gallino; Goya; Grandis; Gravis; I; Jacques; LE; Labarta; Lusso; M; ME; MI; MS; Medina; Mistchenko; N; Nabaes Jodar; Natale; O; Primost; S; Streitenberger; Thomas; Valinotto; Viegas, M.; Villegas
EPI_ISL_1396092, EPI_ISL_1396093, EPI_ISL_1396094, EPI_ISL_1396095, EPI_ISL_1396096, EPI_ISL_1396097, EPI_ISL_1396098, EPI_ISL_1396099, EPI_ISL_1396100, EPI_ISL_1396101, EPI_ISL_1396102, EPI_ISL_1396103, EPI_ISL_1396104, EPI_ISL_1396105, EPI_ISL_1396106, EPI_ISL_1396107, EPI_ISL_1396108, EPI_ISL_1396109, EPI_ISL_1396110, EPI_ISL_1396111, EPI_ISL_1396112, EPI_ISL_1396113, EPI_ISL_1396114, EPI_ISL_1396115, EPI_ISL_1396116, EPI_ISL_1396117, EPI_ISL_1396118, EPI_ISL_1396119, EPI_ISL_1396120, EPI_ISL_1396121, EPI_ISL_1396122, EPI_ISL_1396123			
see above	Laboratorio de Virus Respiratorios y Neurovirosis. Hospital Señor del Milagro	Instituto de Patologia Vegetal (CIAP-INTA) on behalf of 'Proyecto Argentino Interinstitucional de genomica de SARS-CoV-2' (PAIS Consortium)	A. Dra. Raskovsky Viviana; Amadio; Debat, HJ.; Dr. Lavaque Esteban; Dra. Veronica Lesser. Tecnica: Pamela Cajal; FD; Fernanda Aguero.; Fernández; Irazoqui, M.; Marquez, N.
EPI_ISL_792291, EPI_ISL_792292, EPI_ISL_792293, EPI_ISL_792294, EPI_ISL_792295, EPI_ISL_792296, EPI_ISL_792297, EPI_ISL_792298			
see above	Laboratorio de genética y biología molecular del Hospital de trauma y emergencia Dr Federico Abete	Área de Secuenciación del Laboratorio de Virología del Hospital de Niños Dr. Ricardo Gutierrez on behalf of 'Proyecto Argentino Interinstitucional de genomica de SARS-CoV-2' (PAIS Consortium)	Gallino; Goya; I; LE; Lusso; MI; MS; Nabaes Jodar; Natale; Primost; S; Valinotto; Viegas, M.
EPI_ISL_1396124, EPI_ISL_1396125, EPI_ISL_1396126, EPI_ISL_1396127, EPI_ISL_1396128, EPI_ISL_1396129, EPI_ISL_1396130, EPI_ISL_1396131, EPI_ISL_1396140, EPI_ISL_1396141, EPI_ISL_1396142, EPI_ISL_1396143, EPI_ISL_1396144, EPI_ISL_1396145, EPI_ISL_1396146, EPI_ISL_1396147, EPI_ISL_1396148			
see above	Laboratorio de la Dirección de Epidemiología	Área de Secuenciación del Laboratorio de Virología del Hospital de Niños Dr. Ricardo Gutierrez on behalf of 'Proyecto Argentino Interinstitucional de genomica de SARS-CoV-2' (PAIS Consortium)	AG; Acuña; Alexay; Bertone; C; D; Goya; JC; LE; Lusso; M; MI; Nabaes Jodar; Natale; Rechimont; S; Usero; Valinotto; Viegas, M.; Villasana
EPI_ISL_1394922, EPI_ISL_1394923, EPI_ISL_1394924	Laboratorio de salud pública, Facultad de Ciencias Exactas, UNLP	Área de Secuenciación del Laboratorio de Virología del Hospital de Niños Dr. Ricardo Gutierrez on behalf of 'Proyecto Argentino Interinstitucional de genomica de SARS-CoV-2' (PAIS Consortium)	Acuña; Alexay; Andrés Angelletti; Andrés Cordero; Carina Tersigni; D; Goya; LE; Laura Delaplace; Lusso; M; MI; Nabaes Jodar; Natale; Rosana Isabel Toro; S; Valinotto; Victoria Nadalich; Viegas, M.; Víctoria Cabassi
EPI_ISL_792375, EPI_ISL_792376, EPI_ISL_792377, EPI_ISL_792378, EPI_ISL_792379, EPI_ISL_792380, EPI_ISL_792381, EPI_ISL_792382, EPI_ISL_792383, EPI_ISL_792384, EPI_ISL_792385			
see above	Laboratorio de salud pública, Facultad de Ciencias Exactas, Universidad Nacional de La Plata	Área de Secuenciación del Laboratorio de Virología del Hospital de Niños Dr. Ricardo Gutierrez on behalf of 'Proyecto Argentino Interinstitucional de genomica de SARS-CoV-2' (PAIS Consortium)	A; Angeletti; Cordero; Goya; LE; Lusso; MI; MS; Nabaes Jodar; Nadalich; Natale; R; S; Toro; V; Valinotto; Viegas, M.
EPI_ISL_792299, EPI_ISL_792300	Laboratorio del Hospital Alemán	Área de Secuenciación del Laboratorio de Virología del Hospital de Niños Dr. Ricardo Gutierrez on behalf of 'Proyecto Argentino Interinstitucional de genomica de SARS-CoV-2' (PAIS Consortium)	Della Latta; E; García Allende; Goya; Ibañez; LE; Lusso; MI; MP; MS; N; Nabaes Jodar; Natale; S; Valinotto; Viegas, M.
EPI_ISL_792306, EPI_ISL_792307, EPI_ISL_792308, EPI_ISL_792309, EPI_ISL_792310, EPI_ISL_792311, EPI_ISL_792312, EPI_ISL_792313, EPI_ISL_792314, EPI_ISL_792315, EPI_ISL_792316, EPI_ISL_792317, EPI_ISL_792318, EPI_ISL_792319			
see above	Laboratorio del Hospital El Cruce Dr. Néstor C. Kirchner	Área de Secuenciación del Laboratorio de Virología del Hospital de Niños Dr. Ricardo Gutierrez on behalf of 'Proyecto Argentino Interinstitucional de genomica de SARS-CoV-2' (PAIS Consortium)	Goya; LE; Lusso; M; MI; MS; Nabaes Jodar; Natale; Rahhal; S; Valinotto; Viegas, M.; Zubieta
EPI_ISL_1394948, EPI_ISL_1394972, EPI_ISL_1394985, EPI_ISL_1394998, EPI_ISL_1394999, EPI_ISL_1395000, EPI_ISL_1395017, EPI_ISL_1395018, EPI_ISL_1395019, EPI_ISL_1395020, EPI_ISL_1395036, EPI_ISL_1395037, EPI_ISL_1395038, EPI_ISL_1395039, EPI_ISL_1395040, EPI_ISL_1395052, EPI_ISL_1395053, EPI_ISL_1395054, EPI_ISL_1395067, EPI_ISL_1395068, EPI_ISL_1395069			
see above	Laboratorio del Hospital Interzonal General de Agudos "Evita"	Área de Secuenciación del Laboratorio de Virología del Hospital de Niños Dr. Ricardo Gutierrez on behalf of 'Proyecto Argentino Interinstitucional de genomica de SARS-CoV-2' (PAIS Consortium)	Acuña; Alejandra Musto; Alexay; D; Erica Luczak; Goya; Isabel Desimone; LE; Lorena Serrano; Lusso; M; MI; Nabaes Jodar; Natale; Omar Grossi; S; Valinotto; Viegas, M.
EPI_ISL_792371, EPI_ISL_792372, EPI_ISL_792373, EPI_ISL_792374	Laboratorio del Hospital Interzonal General de Agudos Eva Perón	Área de Secuenciación del Laboratorio de Virología del Hospital de Niños Dr. Ricardo Gutierrez on behalf of 'Proyecto Argentino Interinstitucional de genomica de SARS-CoV-2' (PAIS Consortium)	C; Carulla; Goya; Kairiyama; LE; Lusso; M; MI; MS; Nabaes Jodar; Natale; Pengue; Piñeyro C; S; Valinotto; Viegas, M.
EPI_ISL_792525	Laboratorio del Hospital Interzonal General de Agudos Evita	Grupo de Genómica y Bioinformática del Instituto de Investigación de la Cadena Láctea CONICET-INTA on behalf of 'Proyecto Argentino Interinstitucional de genomica de SARS-CoV-2' (PAIS Consortium)	AF; Alexay; Amadio; Aulicino; C; Desimone; E; Eberhardt; G; Goya; Grossi; I; Irazoqui; König; L; Luczac; M; MF; MS; Musto; Nabaes Jodar; O; P; S; Serrano; Torres; Viegas, M.
EPI_ISL_792333, EPI_ISL_792334, EPI_ISL_792335, EPI_ISL_792336, EPI_ISL_792337, EPI_ISL_792338, EPI_ISL_792339, EPI_ISL_792340, EPI_ISL_792341, EPI_ISL_792342, EPI_ISL_792343, EPI_ISL_792344, EPI_ISL_792345, EPI_ISL_792346, EPI_ISL_792347, EPI_ISL_792348, EPI_ISL_792349, EPI_ISL_792350			
see above	Laboratorio del Hospital Interzonal General de Agudos Evita	Área de Secuenciación del Laboratorio de Virología del Hospital de Niños Dr. Ricardo Gutierrez on behalf of 'Proyecto Argentino Interinstitucional de genomica de SARS-CoV-2' (PAIS Consortium)	A; Desimone; E; Goya; Grossi; I; L; LE; Luczac; Lusso; MI; MS; Musto; Nabaes Jodar; Natale; O; S; Serrano; Valinotto; Viegas, M.
EPI_ISL_792443, EPI_ISL_792444, EPI_ISL_792445, EPI_ISL_792446, EPI_ISL_792447, EPI_ISL_792448, EPI_ISL_792450, EPI_ISL_792451, EPI_ISL_792452, EPI_ISL_792453, EPI_ISL_792454, EPI_ISL_792455, EPI_ISL_792456, EPI_ISL_792457, EPI_ISL_792458, EPI_ISL_792459, EPI_ISL_792460, EPI_ISL_792461, EPI_ISL_792462, EPI_ISL_792463, EPI_ISL_792464, EPI_ISL_792465, EPI_ISL_792466, EPI_ISL_792467, EPI_ISL_792468, EPI_ISL_792469, EPI_ISL_792470, EPI_ISL_792471, EPI_ISL_792472, EPI_ISL_792473			
see above	Laboratorio del Hospital Regional Ushuaia Gdor. Ernesto Campos	Hospital Regional Ushuaia - Centro Austral De Investigaciones Científicas - Universidad Nacional De Tierra Del Fuego on behalf of 'Proyecto Argentino Interinstitucional de genomica de SARS-CoV-2' (PAIS Consortium)	Boutureira, MF.; CA; CB; CF; Castro; Ceballos; Cáceres; De Roccis; F; G; Gallego; Gramundi; ID; Nardi; SB; SG; Yulan
EPI_ISL_1396332, EPI_ISL_1396333, EPI_ISL_1396334, EPI_ISL_1396335, EPI_ISL_1396336, EPI_ISL_1396337, EPI_ISL_1396338, EPI_ISL_1396339, EPI_ISL_1396340, EPI_ISL_1396341, EPI_ISL_1396342, EPI_ISL_1396343, EPI_ISL_1396344, EPI_ISL_1396345, EPI_ISL_1396346, EPI_ISL_1396347, EPI_ISL_1396348, EPI_ISL_1396349, EPI_ISL_1396350, EPI_ISL_1396351, EPI_ISL_1396352, EPI_ISL_1396353, EPI_ISL_1396354, EPI_ISL_1396355			
see above	Laboratorio del Hospital Regional Ushuaia Gdor. Ernesto Campos	Nodo de Secuenciación Tierra del Fuego - Hospital Regional Ushuaia - Centro Austral De Investigaciones Científicas - Universidad Nacional De Tierra Del Fuego on behalf of 'Proyecto Argentino Interinstitucional de genomica de SARS-CoV-2' (PAIS Consortium)	Alejandro Ezequiel Rojas; Carina Andrea De Roccis; Carolina Beatriz Yulan; Cristina Fernanda Nardi; Fernando Gallego; Gabriel Alejandro Castro; Ivan Dario Gramundi; Manuel Fabian Boutureira; Santiago Guillermo Ceballos; Silvana Beatriz Cáceres
EPI_ISL_1394916	Laboratorio del Hospital Teresa Germani Laferrere	Área de Secuenciación del Laboratorio de Virología del Hospital de Niños Dr. Ricardo Gutierrez on behalf of 'Proyecto Argentino Interinstitucional de genomica de SARS-CoV-2' (PAIS Consortium)	Acuña; Alexay; Anabel Luzio; D; Gisela Sauco; Goya; LE; Lusso; M; MI; Mauricio Spacaventto; Nabaes Jodar; Natale; S; Valinotto; Viegas, M.
EPI_ISL_792386, EPI_ISL_792387, EPI_ISL_792388, EPI_ISL_792389, EPI_ISL_792390, EPI_ISL_792391, EPI_ISL_792392, EPI_ISL_792393, EPI_ISL_792394, EPI_ISL_792395, EPI_ISL_792396			
see above	Plataforma de Servicios Biotecnólogicos: UTTIPP/PSB , Universidad Nacional de Quilmes.	Área de Secuenciación del Laboratorio de Virología del Hospital de Niños Dr. Ricardo Gutierrez on behalf of 'Proyecto Argentino Interinstitucional de genomica de SARS-CoV-2' (PAIS Consortium)	A; Cardama; Castello; Farina; G; Goya; Goñi; H; LE; Lusso; MI; MS; Nabaes Jodar; Natale; S; Valinotto; Viegas, M.
EPI_ISL_1394910, EPI_ISL_1394917, EPI_ISL_1394918, EPI_ISL_1394919, EPI_ISL_1394920, EPI_ISL_1394921, EPI_ISL_1394936, EPI_ISL_1394937, EPI_ISL_1394938, EPI_ISL_1394939, EPI_ISL_1394940, EPI_ISL_1394945, EPI_ISL_1394946, EPI_ISL_1394949, EPI_ISL_1395041			
see above	Plataforma de Servicios Biotecnólogicos; UTTIPP/PSB	Área de Secuenciación del Laboratorio de Virología del Hospital de Niños Dr. Ricardo Gutierrez on behalf of 'Proyecto Argentino Interinstitucional de genomica de SARS-CoV-2' (PAIS Consortium)	Acuña; Alejandra Zinni; Alejandro Castello; Alexay; Carla Capobianco; D; Georgina Cardama; Goya; Gustavo Bad; Hernán Farina; Humberto Lamdan; LE; Lusso; M; MI; Marcelo Mandile; Nabaes Jodar; Natale; Norailys Lorenzo; S; Sandra Goñi; Valinotto; Viegas, M.
EPI_ISL_648217	SILAB	Laboratorio Mixto de Biotecnología Acuática (LMBA)	Adriana Giri; Agustina Cerri; Ana Cavatorta; Ana Paletta; Diego Chouhy; Elisa Bolatti; Elizabeth Tapia; Federico Remes Lenicov; Flavio Spetale; Gastón Viarengo; Ignacio García Labari; Javier Murillo; Joaquín Ezpeleta; Julian Acosta; Laura Angelone; Leandro Ciappina; María Re; Pablo Casal; Pilar Bulacio; Silvana Spinelli; Silvia Arranz; Sofía Lavista Llanos; Vanina Villanova; Victoria Posner
EPI_ISL_420598, EPI_ISL_420599, EPI_ISL_420600, EPI_ISL_778843, EPI_ISL_849148, EPI_ISL_849149, EPI_ISL_849150, EPI_ISL_849151, EPI_ISL_849152, EPI_ISL_849153, EPI_ISL_849154, EPI_ISL_849155, EPI_ISL_849156, EPI_ISL_849157, EPI_ISL_849158, EPI_ISL_849159, EPI_ISL_849160, EPI_ISL_849161, EPI_ISL_849162, EPI_ISL_849163, EPI_ISL_849164, EPI_ISL_849165, EPI_ISL_849166, EPI_ISL_849167, EPI_ISL_849168, EPI_ISL_849169, EPI_ISL_849170, EPI_ISL_849171, EPI_ISL_849172, EPI_ISL_849173, EPI_ISL_849174, EPI_ISL_849175, EPI_ISL_849176, EPI_ISL_849177, EPI_ISL_849178, EPI_ISL_849179, EPI_ISL_849180, EPI_ISL_849181, EPI_ISL_849182, EPI_ISL_849183, EPI_ISL_849184, EPI_ISL_849185, EPI_ISL_849186, EPI_ISL_849187, EPI_ISL_849188, EPI_ISL_849189, EPI_ISL_849190, EPI_ISL_849191, EPI_ISL_849192, EPI_ISL_849193, EPI_ISL_849194, EPI_ISL_849195, EPI_ISL_849196, EPI_ISL_849197, EPI_ISL_849198, EPI_ISL_849199, EPI_ISL_849200, EPI_ISL_849201, EPI_ISL_849202, EPI_ISL_849203, EPI_ISL_849204, EPI_ISL_849279, EPI_ISL_849280, EPI_ISL_849281, EPI_ISL_849282, EPI_ISL_849283, EPI_ISL_849284, EPI_ISL_849285, EPI_ISL_849286, EPI_ISL_849287, EPI_ISL_849288, EPI_ISL_849289, EPI_ISL_849290, EPI_ISL_849291, EPI_ISL_849292, EPI_ISL_849293, EPI_ISL_849294, EPI_ISL_849295, EPI_ISL_849296, EPI_ISL_849297, EPI_ISL_849298, EPI_ISL_849299, EPI_ISL_849300, EPI_ISL_849301, EPI_ISL_849302, EPI_ISL_849303, EPI_ISL_849304, EPI_ISL_849305, EPI_ISL_849306, EPI_ISL_849307, EPI_ISL_849308, EPI_ISL_849309, EPI_ISL_849310, EPI_ISL_849311, EPI_ISL_849312, EPI_ISL_849313, EPI_ISL_849314, EPI_ISL_849315, EPI_ISL_849316, EPI_ISL_849317, EPI_ISL_849318, EPI_ISL_849319, EPI_ISL_849320, EPI_ISL_849321, EPI_ISL_849350, EPI_ISL_849351, EPI_ISL_849352, EPI_ISL_849353, EPI_ISL_849354, EPI_ISL_849355, EPI_ISL_849356, EPI_ISL_849357, EPI_ISL_849358, EPI_ISL_849359, EPI_ISL_849360, EPI_ISL_849361, EPI_ISL_849362, EPI_ISL_849363, EPI_ISL_849364, EPI_ISL_849365, EPI_ISL_849366, EPI_ISL_849367, EPI_ISL_849368, EPI_ISL_849369, EPI_ISL_849370, EPI_ISL_849371, EPI_ISL_849372, EPI_ISL_849373, EPI_ISL_849374, EPI_ISL_849375, EPI_ISL_849376, EPI_ISL_849377, EPI_ISL_849378, EPI_ISL_849379, EPI_ISL_849380, EPI_ISL_849381, EPI_ISL_849382, EPI_ISL_856756, EPI_ISL_856757, EPI_ISL_856758, EPI_ISL_856759, EPI_ISL_856760, EPI_ISL_856761, EPI_ISL_856762, EPI_ISL_856763, EPI_ISL_856764, EPI_ISL_856765, EPI_ISL_856766, EPI_ISL_856767, EPI_ISL_856768, EPI_ISL_856769, EPI_ISL_856770, EPI_ISL_856771, EPI_ISL_856772, EPI_ISL_856773, EPI_ISL_856774, EPI_ISL_856775, EPI_ISL_856776, EPI_ISL_856777, EPI_ISL_856778, EPI_ISL_856779, EPI_ISL_856780, EPI_ISL_856781, EPI_ISL_856782, EPI_ISL_856783, EPI_ISL_856784, EPI_ISL_856785, EPI_ISL_856786, EPI_ISL_856787, EPI_ISL_856788, EPI_ISL_856789, EPI_ISL_856790, EPI_ISL_856791, EPI_ISL_856792, EPI_ISL_856793, EPI_ISL_856794, EPI_ISL_856795, EPI_ISL_856796, EPI_ISL_856797, EPI_ISL_856798, EPI_ISL_856799, EPI_ISL_856800, EPI_ISL_856801, EPI_ISL_2104757, EPI_ISL_2104758, EPI_ISL_2104759, EPI_ISL_2104760, EPI_ISL_2104761, EPI_ISL_2104762, EPI_ISL_2104763, EPI_ISL_2104764, EPI_ISL_2104765, EPI_ISL_2104766, EPI_ISL_2104767, EPI_ISL_2104768, EPI_ISL_2104769, EPI_ISL_2104770, EPI_ISL_2104771, EPI_ISL_2104772, EPI_ISL_2104773, EPI_ISL_2104774, EPI_ISL_2104775, EPI_ISL_2104776, EPI_ISL_2104777, EPI_ISL_2104778, EPI_ISL_2104779, EPI_ISL_2135134, EPI_ISL_2158693			
see above	Servicio Virosis Respiratorias-Departamento Virología-INEI	Instituto Nacional Enfermedades Infecciosas C.G.Malbran	Avaro M.; Baumeister E.; Benedetti E.; Campos J.; Cisterna D.; Dattero ME; Lorenzo F.; Molina V.; Perandones C.; Poklepovich T.; Pontoriero A.; Russo M.; Tuduri E.
EPI_ISL_964888, EPI_ISL_964889, EPI_ISL_964890, EPI_ISL_964892, EPI_ISL_964897, EPI_ISL_964901, EPI_ISL_964913, EPI_ISL_964915			
see above	Sistema Integrado de Emergencias de Neuquén (SIEN)	Laboratorio Central Mg. Luis Alfredo Pianciola on behalf of 'Proyecto Argentino Interinstitucional de genomica de SARS-CoV-2' (PAIS Consortium)	Antonela De Fino; C Pintos; C Ziehm; Darío Fabián di Prátula; J Ousset; L Pianciola.; Liliana Fonseca; M Fernandez; M Mazzeo; M Nabaes; Marcela Nóbile; María Laura Álvarez; Patricia Valeria Blanco; Silvana Cecchi; Yesica Espasandin
EPI_ISL_792302, EPI_ISL_792303, EPI_ISL_792304, EPI_ISL_792305	Unidad de Virología, Centro de Educación Médica en Investigaciones Clínicas CEMIC	Área de Secuenciación del Laboratorio de Virología del Hospital de Niños Dr. Ricardo Gutierrez on behalf of 'Proyecto Argentino Interinstitucional de genomica de SARS-CoV-2' (PAIS Consortium)	AS; Echavarría; Goya; LE; Lusso; M; MI; MS; Mistchenko; Nabaes Jodar; Natale; S; Valinotto; Viegas, M.
EPI_ISL_1914577	Unidad de diagnóstico COVID-19, Facultad de Ciencias Veterinarias (FCV), Universidad Nacional de La Plata (UNLP), Argentina.	Área de Secuenciación del Laboratorio de Virología del Hospital de Niños Dr. Ricardo Gutierrez on behalf of 'Proyecto Argentino Interinstitucional de	Carlos Javier Panei; David Di Llulo; Fernando David Rivero; Gastón More; Juan Manuel Unzaga; Laura Elena Valinotto; Lorena De Felice; Marcelo Pecoraro; Marcos Salina; Mariana Viegas; María Emilia Bravi; Mercedes Soledad Nabaes Jodar; Nadia Analía Fuentealba

We gratefully acknowledge the following Authors from the Originating laboratories responsible for obtaining the specimens, as well as the Submitting laboratories where the genome data were and shared via GISAID, on which this research is based.
Authors are sorted alphabetically. All submitters of data may be contacted directly via www.gisaid.org.
